# Neuroanatomy and behavior in mice with a haploinsufficiency of AT-rich interactive domain 1B (ARID1B) throughout development

**DOI:** 10.1186/s13229-021-00432-y

**Published:** 2021-03-23

**Authors:** J. Ellegood, S. P. Petkova, A. Kinman, L. R. Qiu, A. Adhikari, A. A. Wade, D. Fernandes, Z. Lindenmaier, A. Creighton, L. M. J. Nutter, A. S. Nord, J. L. Silverman, J. P. Lerch

**Affiliations:** 1 Mouse Imaging Centre (MICe), Hospital for Sick Children, 25 Orde Street, Toronto, ON M5T 3H7 Canada; 2 Department of Psychiatry and Behavioral Sciences, MIND Institute, School of Medicine, University of California, Davis, Sacramento, CA USA; 3 Neuroscience Graduate Group, University of California - Davis, Davis, CA USA; 4 Wellcome Centre for Integrative Neuroimaging, FMRIB, Nuffield Department of Clinical Neuroscience, The University of Oxford, Oxford, UK; 5 Department of Medical Biophysics, University of Toronto, Toronto, ON Canada; 6 The Centre for Phenogenomics, Hospital for Sick Children, Toronto, ON Canada; 7 Department of Neurobiology, Physiology and Behavior, University of California - Davis, Davis, CA USA

**Keywords:** Magnetic resonance imaging, Coffin–Siris syndrome, Autism, Arid1b, Mouse, Behavior

## Abstract

**Background:**

One of the causal mechanisms underlying neurodevelopmental disorders (NDDs) is chromatin modification and the genes that regulate chromatin. AT-rich interactive domain 1B *(ARID1B)*, a chromatin modifier, has been linked to autism spectrum disorder and to affect rare and inherited genetic variation in a broad set of NDDs.

**Methods:**

A novel preclinical mouse model of *Arid1b* deficiency was created and validated to characterize and define neuroanatomical, behavioral and transcriptional phenotypes. Neuroanatomy was assessed ex vivo in adult animals and in vivo longitudinally from birth to adulthood. Behavioral testing was also performed throughout development and tested all aspects of motor, learning, sociability, repetitive behaviors, seizure susceptibility, and general milestones delays.

**Results:**

We validated decreased *Arid1b* mRNA and protein in *Arid1b*^+/−^ mice, with signatures of increased axonal and synaptic gene expression, decreased transcriptional regulator and RNA processing expression in adult *Arid1b*^+/−^ cerebellum. During neonatal development, *Arid1b*^+/−^ mice exhibited robust impairments in ultrasonic vocalizations (USVs) and metrics of developmental growth. In addition, a striking sex effect was observed neuroanatomically throughout development. Behaviorally, as adults, *Arid1b*^+/−^ mice showed low motor skills in open field exploration and normal three-chambered approach. *Arid1b*^+/−^ mice had learning and memory deficits in novel object recognition but not in visual discrimination and reversal touchscreen tasks. Social interactions in the male–female social dyad with USVs revealed social deficits on some but not all parameters. No repetitive behaviors were observed. Brains of adult *Arid1b*^+/−^ mice had a smaller cerebellum and a larger hippocampus and corpus callosum. The corpus callosum increase seen here contrasts previous reports which highlight losses in corpus callosum volume in mice and humans.

**Limitations:**

The behavior and neuroimaging analyses were done on separate cohorts of mice, which did not allow a direct correlation between the imaging and behavioral findings, and the transcriptomic analysis was exploratory, with no validation of altered expression beyond *Arid1b*.

**Conclusions:**

This study represents a full validation and investigation of a novel model of *Arid1b*^+/−^ haploinsufficiency throughout development and highlights the importance of examining both sexes throughout development in NDDs.

**Supplementary Information:**

The online version contains supplementary material available at 10.1186/s13229-021-00432-y.

## Background

Neurodevelopmental disorders (NDDs), including autism spectrum disorder (ASD) and intellectual disability (ID), are prevalent and pervasive lifelong disorders defined exclusively by behavior. While numerous etiologies exist, currently there are no biological markers. The NDD behavioral phenotype is extremely heterogeneous, as are the genetics: The Simons Foundation Autism Research Initiative (SFARI) currently lists over 900 autism-relevant genes (gene.sfari.org) [1, 2], a number that increases if all NDDs are considered. To examine the effect that individual genes have on both the brain and behavior, several groups have systematically generated genetic mouse models with mutations of high-confidence NDD and ASD relevant genes.

Chromatin modification is thought to be one of the causal mechanisms underlying NDDs, as several chromatin-modifying genes are high-confidence genes with deletions found in numerous cases of ASD. Chromatin modifiers account for rare and inherited genetic variation in many NDD cases [3, 4]. Genes that regulate chromatin can modify events regulating the formation of neural connections and are also critical for correct epigenetic marking with histone post-translational modifications [5]. AT-rich interactive domain 1B, *ARID1B*, and genes in the chromatin modification complex, SWItch/Sucrose Non-Fermentable (SWI/SNF), are thought to cause Coffin–Siris syndrome (CSS) [6], an exceedingly rare neurodevelopmental disorder with fewer than 200 individuals diagnosed [7]. Clinical presentation includes developmental delay, identified by difficulties with feeding, low birth weight, and general failure to thrive, along with ID, impaired motor skills, and delayed or absent development of speech and language. Neuroanatomical assessments in the first CSS cases reported smaller head circumferences [6], Dandy Walker malformations (hypoplasia of the cerebellum) [8, 9], and an abnormally thin corpus callosum [10]. In one study, the patient additionally had striking deficits in the brainstem and cerebellum which included ectopic neurons throughout the medulla, inferior olive, and cerebellar white matter [10]. In a recent report of 143 patients with *ARID1B* mutations, agenesis or hypoplasia of the corpus callosum was observed in 43% of patients [11].

Comprehensive, mesoscopic neuroanatomical assessments of NDD mouse models have become frequent [12–18]. However, with over 900 genes linked to NDD, characterization of the more prominent and closely linked genetic models, like *Arid1b*, are imperative. Characterizing the anatomical and functional impact of a germline *Arid1b* mutation on brain development, growth, and behavior in vivo could reveal specific and generalizable mechanisms linking chromatin biology to pathology and outcomes. Having a rigorous reproducible model that allows for testing of both pre- and post-therapeutic interventions may fast forward the therapeutic pipeline.

Three groups have recently published work examining mouse models of *ARID1B* haploinsufficiency [19–21]. Celen et al. reported several behavioral abnormalities relevant to ASD. They also reported neuroanatomical decreases in the dentate gyrus and corpus callosum, consistent with a subset of human patients with CSS [19]. The neuroanatomical focus of the Celen et al. study was only on areas thought to be relevant to CSS, and they did not investigate neuroanatomical differences throughout the brain [19]. The two other *Arid1b* mouse studies also did not investigate whole brain neuroanatomical alterations [20, 21] and focused on smaller microscopic differences in cortical structures. Additionally, all previous studies examined limited windows of time in adulthood missing the developmental window in a gene related to NDDs. Thus, a full behavioral and neuroanatomical assessment across development has not yet been performed.

To address gaps and add rigor to existing reports, we generated a novel preclinical mouse model of *Arid1b* using CRISPR/Cas9 genome engineering and assayed neuroanatomical, behavioral, and transcriptional phenotypes associated with *Arid1b* haploinsufficiency. Behavioral assays were conducted across development, neonatally and in adults, and in vivo manganese-enhanced MRI (MEMRI) was used to assess neuroanatomy across development from birth into adulthood.

## Methods

### Generation of the mice

Precision genetic engineering via the Cas9/CRISPR system was used to generate the *Arid1b* mutant mouse line. This allele from project TCPR0317 was generated at The Centre for Phenogenomics (TCP) by injecting Cas9 nickase (D10A) and single-guide RNAs with spacer sequences of CTGCTTAGCAAGTTACCACT and GCCTGATACAGCACTTACAT targeting the 5′ side and ACACTAAAGGGGTTGCTTTC and CTTGTAATCCCCCTGTAGTA targeting the 3′ side of exon OTTMUSE00000314956 (exon 5) resulting in deletion of Chr17 from 5242523 to 5243410 GRCm38 with insertion of TT. Consistent with earlier studies, this mutation is associated with early embryonic lethality in homozygous carriers. The mouseline C57BL/6N-*Arid1b*^em1(IMPC)Tcp^ was made as part of the NorCOMM2 project funded by Genome Canada and the Ontario Genomics Institute at The Centre for Phenogenomics. It was obtained from the Canadian Mutant Mouse Repository (CMMR).

### **Molecular analyses**

#### **Tissue extraction **

Adult wild-type and heterozygous *Arid1b* mice, of both sexes, were cervically dislocated and brains were rapidly extracted. The cerebral cortex, hippocampus, and cerebellum were dissected and flash frozen over dry ice. Tissue was later homogenized and separated for genomic DNA, RNA, or protein.

### Western blots

Protein was extracted using Pierce RIPA Buffer (ThermoFisher, Waltham, MA) with Protease & Phosphatase Inhibitor Cocktail (ThermoFisher, Waltham, MA). Protein concentrations were measured using Pierce Bicinchoninic acid assay kit (ThermoFisher, Waltham, MA). Forty micrograms of protein lysate was separated on a 4–20% Stacking TGX Gels (BioRad, Hercules, CA) and transferred overnight at 30 V onto Immobilon-FL PVDF membrane (Millipore Sigma, Burlington, MA). PVDF membranes were blocked for 1 h with Intercept Protein-Free Blocking Buffer (LI-COR, Lincoln, NE). Following blocking, PVDF membranes were incubated with ms-Arid1b (1:1000, Abcam, ab47561) and rb-beta actin (1:2000, Millipore Sigma, SAB5600204) in Intercept for 2 h at room temperature. Following incubation, membranes were washed with Tris-buffered saline with Tween (TBST) 3× for 5 min. Membranes were then incubated with Goat anti-rabbit LI-COR 680 (1:2000) and goat anti-mouse LI-COR 800 (1:2000) in Intercept TBS for 1 h at RT. Following incubation, membranes were washed 3× with TBST before storing in 1× TBS. Membranes were imaged on Odyssesy CLx imager (LI-COR, Lincoln, NE). Quantitative analysis was performed using Empiria Studio software (LI-COR, Lincoln, NE).

### qPCR analysis

Tissue was immersed in 350 µl of Trizol and sonicated using a QSonica (QSonica, Newtown, CT). Sonicated tissue was then centrifuged at 10,000 rcf and supernatant was removed and incubated with an equivalent volume of 100% molecular grade ethanol. Total RNA then was extracted following DirectZol RNA Extraction (ZymoSearch, San Diego, CA). cDNA was generated using RevertAid Random Hexamer (ThermoFisher, Waltham, MA). SYBR Green (ThermoFisher, Waltham, MA)-based quantitative real-time PCR was performed using 10 ng of cDNA on a QuantStudio 6 Flex (Applied Biosystems, Foster City, CA). Data are presented as 2–∆∆Ct normalized to *Arid1b*^+/+^. The *Arid1b* primers used were: Forward 5′-GTTGGCTCTCCTGTGGGAAGCAA-3′; Reverse 5′-GTGACTGGCTCAAGGCAGGAT-3′.

### RNA-sequencing

Exploratory transcriptomic analysis was performed as before [12]. Cerebellar tissue was dissected from adult heterozygous *Arid1b*^+/−^ mice and *Arid1b*^+/+^ littermates (postnatal day (PND) 140–150, two samples per genotype, one male and one female). Stranded mRNA sequencing libraries were prepared using a TruSeq Stranded mRNA kit and samples were pooled and sequenced in one lane on the Illumina HiSeq platform using a single-end 100-bp strategy. Each library was quantified and pooled before sequencing at the UC Davis DNA Technologies Core. Reads from RNA-seq were aligned to the mouse genome (mm9) using STAR (version 2.5.3a). Aligned reads mapping to genes were counted at the gene level using subreads featureCounts. The mm9 knownGene annotation track and aligned reads were used to generate quality control information using the full RSeQC tool suite. Unaligned reads were quality checked using FastQC. RNA-seq data is available via GEO.

### Differential expression analysis

Raw count data for all samples were used for differential expression analysis using edgeR. Genes with at least 20 reads per million in at least one sample were included for analysis, resulting in a final set of 8942 genes for differential testing. Tagwise dispersion estimates were generated and differential expression analysis was performed using a generalized linear model with genotype as the variable for testing. Normalized expression levels were generated using the edgeR rpkm function. Normalized log2(RPKM) values were used to plot expression data for *Arid1b*. Pseudocount values were used to plot the summary heatmap. Mouse gene ontology (GO) data was downloaded from Bioconductor (org.Mm.eg.db). We used the goseq package to test for enrichment of GO terms indicating parent:child relationships. For GO analysis, we examined down- and upregulated genes separately for genes meeting an FDR < 0.2. For the enrichment analysis, the test set of differentially expressed genes was compared against the background set of genes expressed here using minimum read-count cutoffs described above.

### **Imaging and Behavior**

#### **Breeding**

All animals were housed in a temperature-controlled vivarium maintained on a 12-h light/dark cycle. All procedures were approved by the Institutional Animal Care and Use Committee either at The Centre for Phenogenomics (TCP) in Toronto, Ontario, Canada, or at the University of California Davis School of Medicine in Sacramento, CA, USA, and were conducted in accordance with the Canadian Council on Animal Care Guide to Care and Use of Experimental Animals or the National Institutes of Health Guide for the Care and Use of Laboratory Animals. Efforts were made to minimize animal suffering and the number of animals used.

Imaging was performed in multiple cohorts of mice. One set was perfusion fixed and examined ex vivo and very high resolution, and another group was examined in vivo longitudinally across development. Both methods provide novel and complementary information about any neuroanatomical differences throughout the brain [22].

### Perfusions

In total, 47 fixed mouse brains were examined: 14 *Arid1b*^+/−^ mice (7 females, 7 males) and 33 *Arid1b*^+/+^ (Wild Type, C57BL/6NCrl, 13 females and 20 males). All mice were perfused at PND60. Mice were anesthetized with a mixture of ketamine/xylazine and intracardially perfused with 30 mL of 0.1 M PBS containing 0.05 U/mL heparin (Sigma) and 2 mM ProHance (Bracco Diagnostics, a Gadolinium contrast agent) followed by 30 mL of 4% paraformaldehyde (PFA) containing 2 mM ProHance [23, 24]. Perfusions were performed with a minipump at a rate of approximately 1 mL/min. After perfusion, mice were decapitated and the skin, lower jaw, ears, and the cartilaginous nose tip were removed. The brain and remaining skull structures were incubated in 4% PFA + 2 mM ProHance overnight at 4 °C then transferred to 0.1 M PBS containing 2 mM ProHance and 0.02% sodium azide for at least 1 month prior to MRI scanning [25].

### Magnetic resonance imaging—ex vivo

A multi-channel 7.0 T MRI scanner (Agilent Inc., Palo Alto, CA) was used to image the brains within their skulls. Sixteen custom-built solenoid coils were used to image the brains in parallel [24, 26]. In order to detect volumetric changes, we used the following parameters for the MRI scan: T2-weighted, 3-D fast spin-echo sequence, with a cylindrical acquisition of k-space, a TR of 350 ms, and TEs of 12 ms per echo for 6 echoes, field-of-view equaled to 20 × 20 × 25 mm^3^ and matrix size equaled to 504 × 504 × 630. Our parameters output an image with 0.040 mm isotropic voxels [27]. The total imaging time was 14 h.

### Mice for longitudinal MRI

Mice were generated by crossing *Arid1b*^+/−^ with a C57BL/6NCrl mice to produce heterozygous offspring. The number of mouse pups in each litter was reduced to 6 to ensure equal manganese intake by pups through maternal milk. On PND2, mice were tattooed with green ink on their paws for identification and tail clipped for genotyping. Male and female mice were scanned longitudinally in two cohorts over 8 different time-points: PND4, 6, 8, 10, 17, 23, 35, and 60. Cohort 1 was scanned over the first five timepoints, and cohort two was scanned over all 8 timepoints to extend our developmental window to PND60. Cohort 1 consisted of 11 males and 11 females, 5 of which were mutants and 6 of which were *Arid1b*^+/+^ controls of each sex. Cohort 2 consisted of 18 males and 18 females, 9 of which were mutants and 9 of which were *Arid1b*^+/+^ controls of each sex. For the first five developmental timepoints, group sizes ranged from 12 to 19 mice per sex per genotype; for the final 3 timepoints group sizes ranged from 7 to 9 mice per sex per genotype. An incomplete group at a given timepoint was due to a loss of either the scan (corruption, movement, etc.) or the animal itself during the longitudinal time-course.

### Magnetic resonance imaging—in vivo

Findings in the ex vivo experiments, and the fact that *Arid1b* haploinsufficiency is relevant to NDDs, highlighted the need to examine the developmental time course of the neuroanatomical differences. Therefore, a longitudinal study was performed to track the development of the neuroanatomy from birth to adulthood, similar to previous work [28, 29]. 24 h prior to the scan, a 0.4 mmol/kg dose of 30 mM manganese chloride (MnCl_2_) was administered as a contrast agent. For mice that were 10 days old or younger, the dam was intraperitoneally injected with MnCl_2_, and pups received MnCl_2_ through maternal milk. Mice that were older than ten days received intraperitoneal injections of MnCl_2_. Up to four mice were scanned simultaneously. Custom built 3D printed holders, which allowed for anesthetic delivery and scavenging, as well as heating were used. During the scan, mice were anesthetized with 1–2% isoflurane, and respiratory rate was monitored using a self-gated signal from a modified 3D gradient echo sequence [30].

A multi-channel 7.0 T MRI scanner with a 30 cm diameter bore (Bruker, Billerica, Massachusetts), equipped with 4 individual cryogenically cooled coils was used to acquire images of the mouse brains. Parameters of the scan are as follows: T1 weighted FLASH 3D gradient echo sequence, TR = 26 ms, TE = 8.250 ms, flip angle = 23°, field of view = 25 × 22 × 22 mm, with a matrix size of 334 × 294 × 294 yielding an isotropic imaging resolution of 75 μm. Imaging time was 58 min. After imaging, mice were transferred to a heated cage for 5–10 min to recover from the anesthesia, and then returned to their home cage.

#### Imaging registration and analysis

Image registration is necessary for quantifying the anatomical differences across images. For both the in vivo and ex vivo images the registration consisted of both linear (rigid then affne) transformations and non-linear transformations. These registrations were performed with a combination of mni_autoreg tools [31] and ANTS (advanced normalization tools) [32, 33]. A two-level registration pipeline was used on the in vivo images and has been detailed in previous studies [28]. Briefly, images from the same age were registered together in the first level creating a consensus average for the brain at each age. For example, all of the PND4 images, both *Arid1b*^+/−^ and *Arid1b*^+/+^, were registered together to create a PND4 average brain, all of the PND6 images are registered together to create a PND6 average brain, etc. In the second level, each age-consensus average was registered to the average of the next-oldest age (i.e., PND4 average registered to the PND6 average, PND6 to the PND8, etc.) Transformations from each level were then concatenated so that every single image in the longitudinal dataset could be mapped to the final PND60 average. For the ex vivo image registration, a simpler one-level registration pipeline is used. After registration, all scans were resampled with the appropriate transform and averaged to create a population atlas representing the average anatomy of the study sample. The result of these registrations were deformation fields that transform images to a consensus average. Therefore, these deformations fields quantify anatomical differences between images.

As detailed in previous studies [34, 35], the Jacobian determinants of the deformation fields were computed and analyzed to measure the volume differences between subjects at every voxel. A pre-existing classified MRI atlas was warped onto the population atlas (containing 282 different segmented structures encompassing cortical lobes, large white matter structures such as the corpus callosum, ventricles, cerebellum, brain stem, and olfactory bulbs [28, 36–39]) to compute the volume of brain structures in all the input images. A linear model with a genotype predictor was used to assess the significance of genotype. The model was either fit to the volume of every structure independently (structure-wise statistics) of fit to every voxel independently (voxel-wise statistics), and multiple comparisons in this study were controlled for using the False Discovery Rate [40].

For the longitudinal analysis, a linear mixed effects model was fit to the volume of every structure with fixed effects of sex, genotype, age, sex–genotype interaction, sex–age interaction, genotype–age interaction, sex–genotype–age interaction, and whole-brain volume; and a random intercept for each mouse. To assess the significance of an effect, a reduced model (model without the particular predictor) was fit to the data and the log likelihood ratio test was used to assess whether the particular effect was significantly important in predicting the data. For example, to evaluate the genotype effect, the reduced model contained fixed effects of sex, age, and sex-age interaction, and significant log likelihood ratio test for a structure results indicate genotype had a notable effect on the volume of that structure. To estimate direction of effect, Satterthwaite approximation [41] was used to estimate degrees of freedom.

#### Behavioral testing

Behavioral experiments were performed on separate cohorts of mice at UC Davis. Cohort 1 (*Arid1b*^+/−^* N* = 28, *Arid1b*^+/+^ *N* = 25) was tested through either ultrasonic vocalizations or postnatal developmental physical milestones and neurological reflexes PND2-14, and then they were again tested through adult behaviors consisting of elevated plus maze, light ↔ dark exploration, open field arena exploration, novel object recognition, self-grooming, three-chambered social approach, male female dyad social interactions, fear conditioning, hot plate and grip strength starting at 10 weeks of age. Cohort 2 (*Arid1b*^+/−^* N* = 24, *Arid1b*^+/+^ *N* = 19) was tested through either ultrasonic vocalizations or developmental milestones PND2-14 and were tested through touchscreen starting at 8 weeks of age. Lethal dose of PTZ served as the endpoint for both cohorts at 18 weeks of age. To minimize the carryover effects from repeated testing, assays were performed in the order of least to the most stressful tasks. Both sexes were used in equal numbers unless otherwise stated such as for the male–female social reciprocal interaction assay.

#### Developmental milestones

Developmental milestones were measured on PND2-14, similar to those previously described [42, 43], but elaborated based on the unique features of this line. All measures were conducted by an experimenter blind to genotype and sex. Pups were separated from the dam for a maximum time of 4 min. Pups were observed for abnormalities in eye, pinnae, whisker, and fur development each testing day. Pups were also picked up to observe limb movement for early signs of hypotonia. Physical feature measurements such as body weight and length (subtraction of total length from tip of nose to base of tail and tail length), and head width (at widest point of head from ear to ear) was measured using a scale (grams) and a digital caliper (mm). Developmental motor milestones to detect neonatal hypotonia and ataxia included negative geotaxis, cliff avoidance, righting reflex and circle transverse. Negative geotaxis was tested by placing each pup, facing downwards, on a surface angled at 45° from parallel, and measuring the time for it to completely turn 180° and face up the incline. Failures to turn and climb were recorded as a maximum score of 30-s. Cliff avoidance was tested by gently placing each pup near the edge of a cardboard box and measuring the time for it to turn its head or back away from the edge. Failures to avoid the cliff was recorded as a maximum score of 30-s. Righting reflex was tested by placing each pup on its back, releasing it, and measuring the time for it to fully flip over onto four paws on each developmental day. Righting reflex, negative geotaxis and cliff aversion all test early limb coordination, vestibular system and proprioception. Circle transverse was tested by placing each pup in the center of a circle with a 5″ (12.5 cm) diameter drawn on a laminated sheet of 8.5″ × 11″ white paper and measuring the time for it to exit the circle. Circle transverse is an early metric of motor ability and ambulation; developmental delay in crawling and walking may be observed using this task. Failures to exit the circle were recorded as a maximum score of 30-s. Milestones for tracking development of fore and hind limb strength were forelimb grasp, forelimb bar holding, forelimb hang, and hindlimb hang. Forelimb grasp tests a mouse’s ability to curl its paw towards an object, a thin wooden stick. Pups typically develop this ability by PND5 and day of first presentation is noted for each pup. Starting around PND6, pups develop enough limb strength to hold themselves up by the forelimbs. Pups are placed by the forelimbs on an unfolded paper clip and time spent able to hang on independently, up to 30 s, is recorded. Hindlimb strength testing can be performed earlier than forelimb strength. Once pups develop some limb strength and the grasp reflex, a thin unfolded paper clip bar is placed into their paws and time able to independently hold the bar, up to 30 s, is recorded. This task requires high compliance and tested starting at PND8. The delay in testing from PND2 to PND8 is the result of this skill’s difficulty to assess. Subjects must grasp, comply for a set amount of time and hold the bar. Hindlimb hang is performed by placing the pup on the thin edge of a surface or container by its legs and allowing the pup to hang down and support itself on the edge by its hindlimbs. Time spent independently hanging by its hindlimbs, up to 30 s, is recorded. Strength tests allow for the determination if motor deficits are due to peripheral limb weakness or centralized neural coordination dysfunction.

#### Ultrasonic vocalizations

Pups were removed individually from the nest at random and gently placed into an isolation container (8 cm × 6 cm × 5 cm; open surface) made of glass. The isolation container was surrounded by a sound attenuating box (18 cm × 18 cm × 18 cm) made of 4 cm thick styrofoam walls. Ultrasonic vocalizations were monitored by an UltraSound Gate Condenser Microphone CM 16 (Avisoft Bioacoustics, Berlin, Germany) attached to the roof of the sound attenuating box, 7 cm above the floor. The microphone was connected via an UltraSoundGate 116 USB audio device (Avisoft Bioacoustics) to a personal computer where acoustic data was recorded with a sampling rate of 250,000 Hz by Avisoft RECORDER (version 2.97; Avisoft Bioacoustics). At the end of the 3-min recording session, both body weight and body temperature were collected. The isolation container was cleaned with 70% ethanol before the beginning of the first test session and between each new litter. Ultrasonic vocalization spectrograms were displayed using Avisoft software and calls were identified manually by a highly trained investigator blinded to genotype.

#### Elevated plus-maze

Conflict anxiety-like behavior via the elevated plus maze was measured according to previously described procedures [44, 45] using a standard mouse apparatus (Med Associates, St. Albans, VT). The maze had two open arms (35.5 cm × 6 cm) and two closed arms (35.5 cm × 6 cm) radiating from a central area (6 cm × 6 cm). A 0.5-cm-high lip surrounded the edges of the open arms. 20 cm high walls enclosed the closed arms. The apparatus was cleaned with 70% ethanol before the beginning of the first test session and after each subject mouse. Room illumination was ~ 300 lx.

#### Light ↔ dark transitions

Light ↔ dark exploration was measured according to previously published procedures [44, 45]. Subject mice were placed in the brightly illuminated, large chamber. The smaller dark chamber (~ 5 lx) was entered by traversing a partition between the two chambers. Subject mice freely explored for 10 min. Time in the dark side chamber and total number of transitions between the light and dark side chambers were automatically recorded using LabVIEW 8.5.1 software (National Instruments, Austin, TX). Room illumination was ~ 400 lx.

#### Open field

General exploratory locomotion in a novel open field environment was assayed as previously described [44, 45]. Open field activity was considered an essential control for effects on physical activity, e.g., sedation or hyperactivity, which could confound the interpretation of results from the reciprocal interactions, self-grooming, novel object recognition, fear conditioning and social approach tasks [46, 47]. The testing room was illuminated at ~ 30 lx.

#### Gait

Treadmill gait analysis was performed using the DigiGait™ system (Mouse Specifics Inc., USA) [48]. Mouse paws were painted with non-toxic red food coloring to augment dark green paw tattoos that generated conflict in DigiGait™ analysis 1 min prior to introduction to the walking chamber to reliably capture the entire paw surface area. Before data collection, each subject was acclimated to the Perspex walking chamber for 1 min and the treadmill was slowly accelerated to the final speed of 20 cm/s to allow mice to adjust to walking on the belt. Digital images of paw placement were recorded through a clear treadmill from the ventral plane of the animal. Mice were tested in a single session at a 20 cm/s treadmill speed maintaining a normal pace walk for *Arid1b*^+/+^ mice. Non-performers were defined as mice who were unable to sustain walking at 20 cm/s without colliding with the posterior bumper for at least 3 s. There is no practice effect or repeated exposure, and therefore, mice were allowed retrial and retest if they were unable to adjust to walking on the belt easily. The treadmill belt and the encasing Perspex chamber were cleaned with 70% (v/v) ethanol in between tests. For each mouse, videos of ∼5 s duration of all sessions were analyzed using the DigiGait™ Imaging and Analysis software v12.2 (Mouse Specifics Inc., USA). Contrast filters were determined on a mouse-by-mouse case to facilitate consistent recognition of all four paws. All analysis was conducted in a single session by experimenter blind to genotype. Stride length (distance a paw makes during a single stride) and frequency (number of strides per second to maintain pace) were automatically calculated. Data was averaged between left and right paws for fore and hind paws.

#### Novel object recognition

The novel object recognition (NOR) test was conducted in opaque matte white (P95 White, Tap Plastics, Sacramento, CA) open field arenas (41 cm × 41 cm × 30 cm), using methods similar to those previously described [12, 43, 45]. The experiment consisted of four sessions: a 30-min exposure to the open field arena the day before the test, a 10-min re-habituation on test day, a 10-min familiarization session and a 5-min recognition test. On day 1, each subject was habituated to a clean, empty open field arena for 30 min. Twenty-four hours later, each subject was returned to the open field arena for another 10 min for the habituation phase. The mouse was then removed from the open field and placed in a clean temporary holding cage for approximately 2 min. Two identical objects were placed in the arena. Each subject was returned to the open field in which it had been habituated and allowed to freely explore for 10-min. After the familiarization session, subjects were returned to their holding cages, which were transferred from the testing room to a nearby holding area. The open field was cleaned with 70% ethanol and let dry. One clean familiar object and one clean novel object were placed in the arena, where the two identical objects had been located during in the familiarization phase. 60-min after the end of the familiarization session, each subject was returned to the arena for a 5-min recognition test, during which time it could to freely explore the familiar object and the novel object. The familiarization session and the recognition test were recorded and scored with Ethovision XT videotracking software (Version 9.0, Noldus Information Technologies, Leesburg, VA). Object investigation was defined as time spent sniffing the object when the nose was oriented toward the object and the nose–object distance was 2 cm or less. Recognition memory was defined as spending significantly more time sniffing the novel object compared to the familiar object via a Student’s paired *t* test. Total time spent sniffing both objects was used as a measure of general exploration. Time spent sniffing two identical objects during the familiarization phase confirmed the lack of an innate side bias. Objects used were plastic toys: a small soft plastic orange safety cone and a hard, plastic magnetic cone with ribbed sides, as previously described [49].

#### Repetitive self-grooming

Spontaneous repetitive self-grooming behavior was scored as previously described [50, 51]. Each mouse was placed individually into a standard mouse cage, (46 cm length × 23.5 cm wide × 20 cm high). Cages were empty to eliminate digging in the bedding, which is a potentially competing behavior. The room was illuminated at ~ 40 lx. A front-mounted CCTV camera (Security Cameras Direct) was placed at ~ 1 m from the cages to record the sessions. Sessions were videotaped for 20 min. The first 10-min period was habituation and was unscored. Each subject was scored for cumulative time spent grooming all the body regions during the second 10 min of the test session. Scoring was conducted by an experimenter blind to genotype.

#### Social approach

Social approach was tested in an automated three-chambered apparatus using methods similar to those previously described [52–54]. Automated Ethovision XT videotracking software (Version 9.0, Noldus Information Technologies, Leesburg, VA) and modified non-reflective materials for the chambers were employed to maximize throughput. The updated apparatus (40 cm × 60 cm × 23 cm) was a rectangular, three-chambered box made from matte white finished acrylic (P95 White, Tap Plastics, Sacramento, CA). Opaque retractable doors (12 cm × 33 cm) were designed to create optimal entryways between chambers (5 cm × 10 cm), while providing maximal manual division of compartments. Three zones, defined using the EthoVision XT software, detected time in each chamber for each phase of the assay. Zones were defined as the annulus extending 2 cm from each novel object or novel mouse enclosure (inverted wire cup, Galaxy Cup, Kitchen Plus, http:// www.kitchen-plus.com). Direction of the head, facing toward the cup enclosure, defined sniff time. A top-mounted infrared-sensitive camera (Ikegami ICD-49, B&H Photo, New York, NY) was positioned directly above every two 3-chambered units. Infrared lighting (Nightvisionexperts.com) provided uniform, low level illumination. The subject mouse was first contained in the center chamber for 10 min, then explored all three empty chambers during a 10-min habituation session, then explored the three chambers containing a novel object in one side chamber and a novel mouse in the other side chamber. Lack of innate side preference was confirmed during the initial 10 min of habituation to the entire arena. Novel stimulus mice were 129 Sv/ImJ, a relatively inactive strain, aged 10–14 weeks, and matched to the subject mice by sex. Number of entries into the side chambers served as a within-task control for levels of general exploratory locomotion.

#### Male female social dyad interaction

The male–female social dyad interaction test was conducted as previously described [45, 51, 53]. Briefly, each freely moving male subject was paired for 5-min with a freely moving unfamiliar estrous *Arid1b*^+/+^ female. A closed-circuit television camera (Panasonic, Secaucus, NJ, USA) was positioned at an angle from the Noldus PhenoTyper arena (Noldus, Leesburg, VA) for optimal video quality. An ultrasonic microphone (Avisoft UltraSoundGate condenser microphone capsule CM15; Avisoft Bioacoustics, Berlin, Germany) was mounted 20 cm above the cage. Sampling frequency for the microphone was 250 kHz, and the resolution was 16 bits. The entire apparatus was contained in a sound-attenuating environmental chamber (Lafayette Instruments, Lafayette, IN) under red light illumination (~ 10 lx). Duration of nose-to-nose sniffing, nose-to-anogenital sniffing, and following were scored using Noldus Observer 8.0XT event recording software (Noldus, Leesburg, VA) as previously described [55]. Ultrasonic vocalization spectrograms were displayed using Avisoft software and calls were identified manually by a highly trained investigator blinded to genotype.

#### Fear conditioning

Delay contextual and cued fear conditioning was conducted using an automated fear-conditioning chamber (Med Associates, St Albans, VT, USA) as previously described [45]. The conditioning chamber (32 × 25 × 23 cm^3^, Med Associates) was interfaced to a PC installed with VideoFreeze software (version 1.12.0.0, Med Associates) and enclosed in a sound-attenuating cubicle. Training consisted of a 2-min acclimation period followed by three tone-shock (CS–US) pairings (80 dB tone, duration 30 s; 0.5 mA footshock, duration 1 s; intershock interval 90 s) and a 2.5-min period, during which no stimuli were presented. The environment was well lit (~ 100 lx), with a stainless-steel grid floor and swabbed with vanilla odor cue (prepared from vanilla extract; McCormick; 1:100 dilution). A 5-min test of contextual fear conditioning was performed 24 h after training, in the absence of the tone and footshock, but in the presence of 100 lx overhead lighting, vanilla odor and chamber cues identical to those used on the training day. Cued fear conditioning, conducted 48 h after training, was assessed in a novel environment with distinct visual, tactile and olfactory cues. Overhead lighting was turned off. The cued test consisted of a 3-min acclimation period followed by a 3-min presentation of the tone CS and a 90-s exploration period. Cumulative time spent freezing in each condition was quantified by VideoFreeze software (Med Associates).

#### Touchscreen pairwise discrimination

Pairwise visual discrimination was tested in the automated Bussey–Saksida touchscreen apparatus for mice (Campden Instruments Ltd/Lafayette Instruments, Lafayette, IL, USA), using a procedure modified from original methods described previously [54, 56–58]. The reinforcer was 20 ul of a palatable liquid nutritional supplement (Strawberry Ensure Plus, Abbott, IL, USA) diluted to 50% with water. Each session was conducted under overhead lighting (~ 60 lx). A standard tone cue was used to signal the delivery of the reinforcer during pre-training and acquisition. Prior to pre-training, subject mice were weighed, and placed on a restricted diet of 2–4 g of rodent chow per mouse per day, to induce a 15% weight loss. Body weight was carefully monitored throughout the experiment, to ensure that a minimum of 85% of free feeding body weight was maintained for each mouse. The pre-training consisted of four stages as previously published [54]. Stage 1 consisted of two days of habituation (20 min on day 1, and 40 min on day 2) to the chamber and the liquid diet with no images on the screen under overhead lighting (~ 60 lx). Stage 2 was a single 45-min session in which entering and exiting the food magazine initiates the next trial and triggers additional reward under overhead lighting. During Stage 3, subjects were trained in daily 45-min sessions during which an image (a random picture from a selection of 40 images) was presented in one of the two windows and remained on the screen until it was touched. Mice must complete 30 trials/day for two consecutive days in order to advance to the next stage. In Stage 4, subjects were trained in 45-min daily sessions in which touching the blank side of the screen was discouraged with a 5-s timeout during which the overhead lighting turned off. Completion of at least 30 trials, at an average accuracy of 80%, on two consecutive days, is required for advancement. Percent accuracy during a session is defined as (# correct trials/# total trials) * 100. Images used in Stages 3 and 4 were not used in the subsequent discrimination task. Only mice that completed all stages of pre-training were advanced to the pairwise visual discrimination task. Subjects were trained to discriminate between two novel images, a spider and an airplane, presented in a spatially pseudo-randomized manner in the two windows of the touchscreen. Each 45-min session consisted of unlimited number of trials separated by 15-s intertrial intervals (ITI). Designation of the correct and incorrect images was counterbalanced across mice within each genotype. Correct responses were rewarded. Each incorrect response was followed by a correction trial in which the images were presented in an identical manner to the previous trial, until a correct response was made. Criterion was completing at least 30 trials, at an accuracy of 80% or higher, on two consecutive days. Days to reach criterion and percentage of mice reaching criterion on each day were compared between genotypes. After successful completion of the task, mice underwent task reversal in which the opposite image was now correct. Criterion was completing at least 30 trials, at an accuracy of 75% or higher, on two consecutive days. Days to reach criterion and percentage of mice reaching criterion on each day were compared between genotypes.

#### Pentylenetetrazol-induced seizures

Behavioral assessment of seizure threshold in mice was performed with injections of 80 mg/kg of pentylenetetrazol (PTZ) as described previously [45, 59]. PTZ-induced convulsions were used to gauge susceptibility to primary generalized seizures and as a gross approximation of excitation–inhibitory balance.

#### Behavioral analysis

Data were analyzed in GraphPad PRISM 7.0. Sexes were considered separately with genotype as the fixed factor. All significance levels were set at *p* < 0.05 and all tests were two-tailed.

#### Experimental design and statistical analysis

All experiments reported in this study were designed to examine genotype effects between the *Arid1b*^+/−^ mice and the *Arid1b*^+/+^ wild-type control (C57BL/6NCrl) mice. In addition, for the longitudinal developmental comparisons, we looked at genotype, sex, age, and the interactions between those factors using a linear mixed effects model described above.

**Fig. 1:**
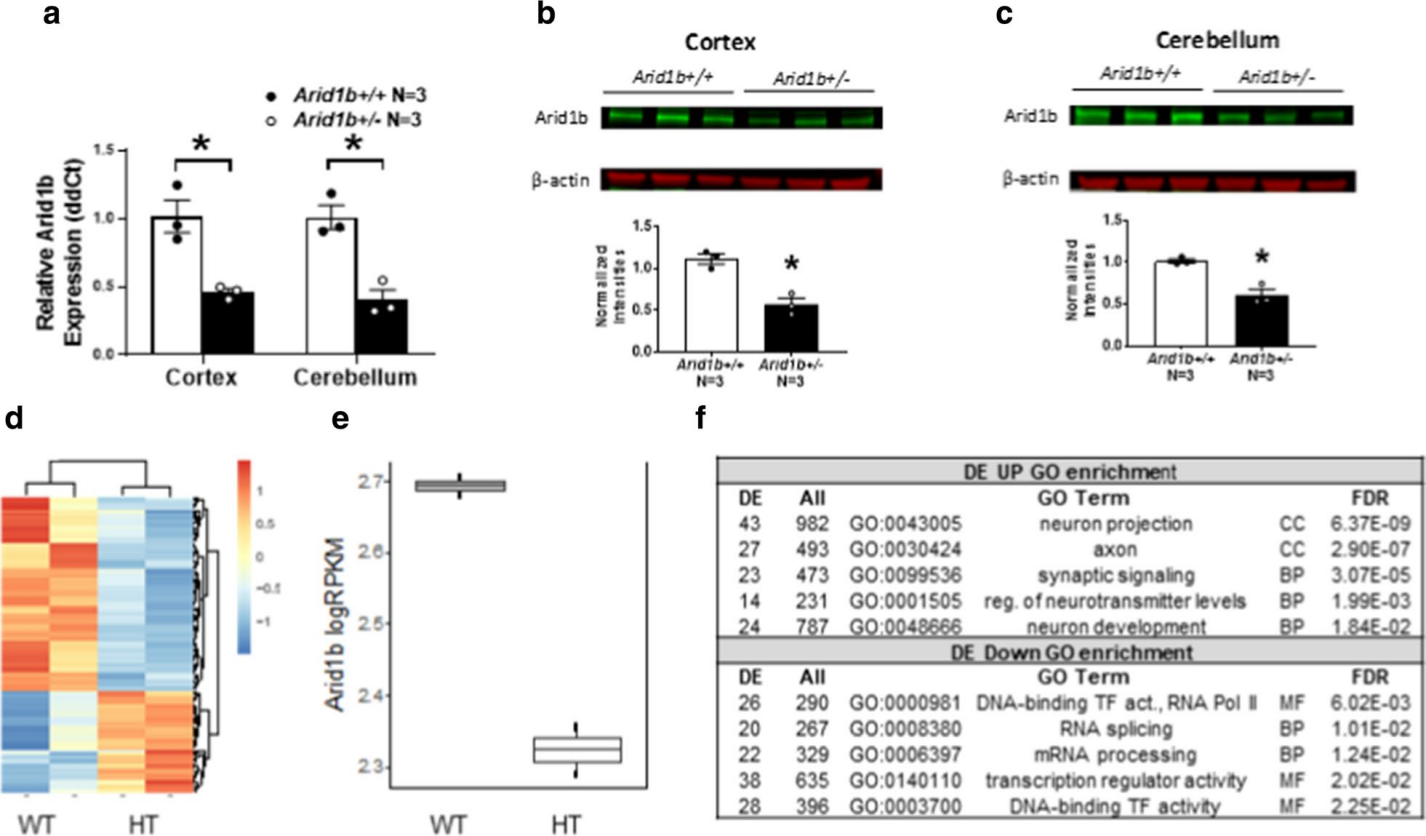
*Arid1b* model validation and differential expression in adult cerebellum. **a** Quantitative reverse-transcription PCR (qRT-PCR) showed reduced mRNA levels of *Arid1b* in *Arid1b*^+/−^ mice compared to wildtype in cortex and cerebellum lysates. Western blot quantitation showed significantly lower relative expression of ARID1B in both the **b** cortex and **c** cerebellum of *Arid1b*^+/−^ mice compared to wildtype. For **a**–**c** *Arid1b*^+/+^ *N* = 3, *Arid1b*^+/−^* N* = 3, mixed sex. *p < 0.05, Student’s *t* test. Error bars represent mean ± SEM. **d** Heatmap showing relative expression in *Arid1b*^+/+^ and *Arid1b*^+/−^ (HT) adult cerebellum for 72 differentially expressed (DE) genes (EdgeR GLM FDR < 0.05, *N* = 1 male and female each per genotype). **e** Bar plot showing logRPKM *Arid1b* expression. **f** Representative GO terms enriched among DE genes (FDR < 0.2).DE and All: DE and total gene count annotated to GO term. GO Term: GO accession, description, and ontology class (Biological Pathway (BP), Molecular Function (MF), Cellular Component (CC)). FDR: goseq FDR for enrichment

For Fig. 1, Transcriptomic analysis was performed as before [12]. Cerebellar tissue was micro-dissected from *Arid1b*^+/−^ mice and *Arid1b*^+/+^ littermates (postnatal day (PND) 140–150). For differential expression, genes with at least 20 reads per million in at least one sample were included for analysis, resulting in a final set of 8942 genes for testing. Normalized log2(RPKM) values were used to plot expression data for *Arid1b*. Pseudo-count values were used to plot the summary heatmap.

**Fig. 2:**
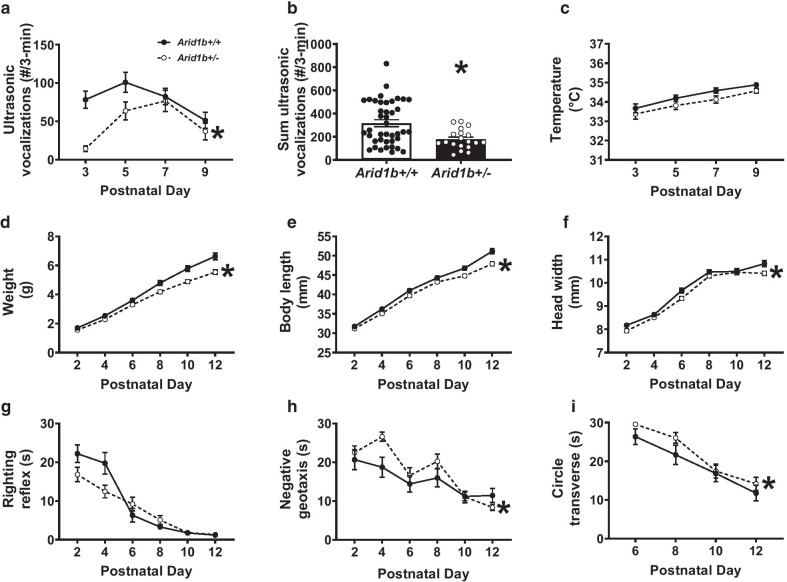
Neonatal ultrasonic vocalization emissions, developmental growth, neurological reflexes and developmental delays in *Arid1b*^+/−^. **a** Across neonatal developmental life, *Arid1b*^+/−^ pups displayed abnormal ultrasonic vocalization (USVs) emissions including delay of peak number of calls and decreased total number of USVs on several days compared to *Arid1b*^+/+^. **b** When summed, *Arid1b*^+/−^ pups emitted significantly fewer USVs compared to *Arid1b*^+/+^ littermate controls. **c** No genotype difference in axillary abdominal temperature was found, confirming fewer USVs were not the result of neonatal hypothermia. **d** *Arid1b*^+/−^ weighed less across development beginning at postnatal 8 and continuously throughout development compared to sex-matched *Arid1b*^+/+^. **e** Across neonatal developmental life, *Arid1b*^+/−^ pups were shorter by body length (**f**) and narrower by head width measurements. **g** While *Arid1b*^+/−^ showed normal latencies to perform the righting reflex, a measure of limb coordination in early life, **h** *Arid1b*^+/−^ had prominent deficits in negative geotaxis (i.e., incline reorientation) and **i** the ability to traverse out of circle by walking. For **a**–**c**, *Arid1b*^+/+^ *N* = 40, *Arid1b*^+/−^* N* = 19, for **d**–**i** *Arid1b*^+/+^ *N* = 29, *Arid1b*^+/+^ *N* = 18. * *p* < .05 versus *Arid1b*^+/+^ by repeated measures two-way ANOVA or student’s unpaired *t* test. Error bars represent mean ± SEM

**Fig. 3:**
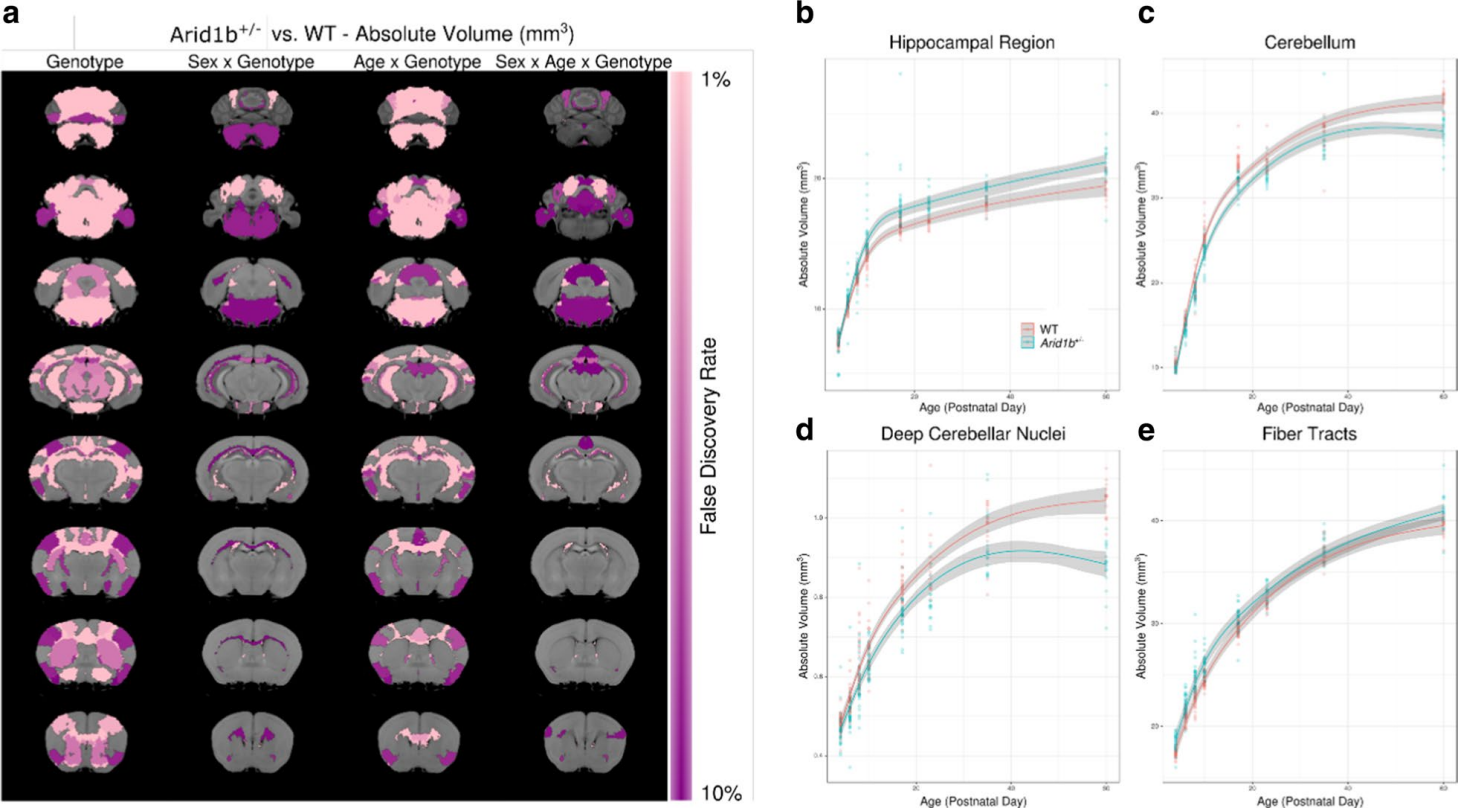
Effects of *Arid1b*^+/−^ on the Brain Throughout Development. **a** Highlights the results from the linear mixed effects model. The first column represents the main effect of genotype across the study sample. The next highlights the sex by genotype interaction, followed by the age by genotype interaction, and the last column shows the three-way interaction between age, sex, and genotype. The growth rates of the **b** hippocampal region, **c** cerebellum, **d** deep cerebellar nuclei, and **e** fiber tracts are shown throughout development. The trend lines shown and the 95% confidence intervals are based on predictions from the linear mixed effects model

**Fig. 4:**
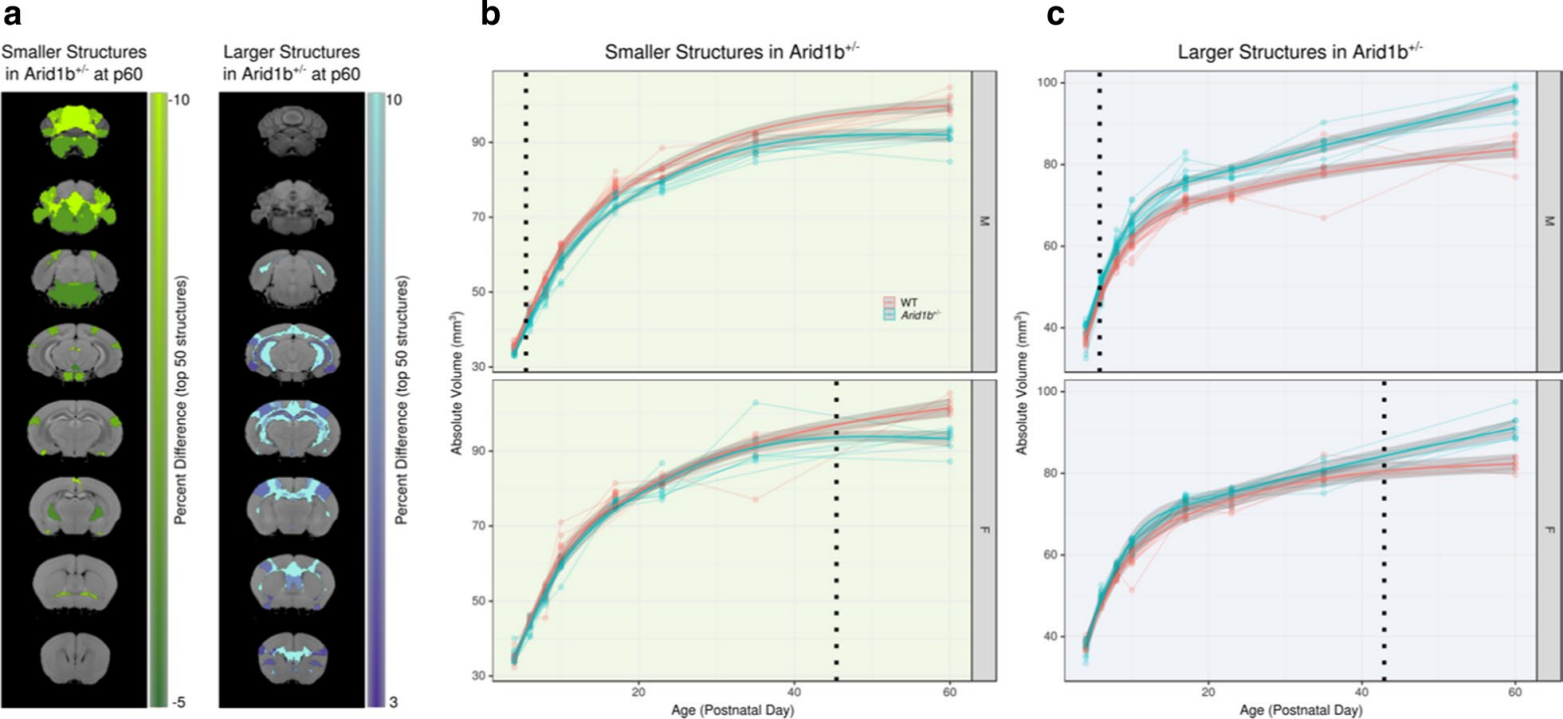
The effect of *Arid1b*^+/−^ on the brain differs between sexes. **a** Highlights the top 50 structures on coronal slices that were larger or smaller in the *Arid1b*^+/− ^mouse at PND60. The delayed emergence of the differences in the female *Arid1b*^+/−^ mutants are shown for the smaller (**b**) and larger (**c**) structures. The dotted line shows when significant differences emerge for the *Arid1b*^+/−^ mice versus the *Arid1b*^+/+^. The trend lines and the 95% confidence intervals shown are based on predictions from the linear mixed effects model

For Fig. 2, ex vivo brains samples from 47 mice were examined: 14 *Arid1b*^+/−^ mice (7 females, 7 males) and 33 *Arid1b*^+/+^ (C57BL/6NCrl, 13 females and 20 males). All mice were perfused at PND60. For Figs. 3 and 4, Male and female mice were scanned longitudinally in two cohorts over 8 different time-points: PND4, 6, 8, 10, 17, 23, 35, and 60. Cohort 1 was scanned over the first five timepoints, and cohort two was scanned over all 8 timepoints to extend our developmental window to PND60. Cohort 1 consisted of 11 males and 11 females, 5 of which were mutants and 6 of which were *Arid1b*^+/+^ controls of each sex. Cohort 2 consisted of 18 males and 18 females, 9 of which were mutants and 9 of which were *Arid1b*^+/+ ^controls of each sex. For the first five developmental timepoints, group sizes ranged from 12 to 19 mice per sex per genotype; for the final 3 timepoints group sizes ranged from 7 to 9 mice per sex per genotype. For calculating the volumes in Figs. 2, 3, and 4, as detailed in previous studies [34, 35], the Jacobian determinants of the deformation fields were computed and analyzed to measure the volume differences between subjects at every voxel. A linear model with a genotype predictor was used to assess the significance of genotype in the ex vivo cohort. The model was either fit to the volume of every structure independently (structure-wise statistics) of fit to every voxel independently (voxel-wise statistics), and multiple comparisons in this study were controlled for using the False Discovery Rate [40]. For the longitudinal cohorts, a linear mixed effects model was fit to the volume of every structure with fixed effects of sex, genotype, age, sex–genotype interaction, sex–age interaction, genotype–age interaction, sex–genotype–age interaction, and whole-brain volume; and a random intercept for each mouse. To assess the significance of an effect, a reduced model (model without the particular predictor) was fit to the data and the log likelihood ratio test was used to assess whether the particular effect was significantly important in predicting the data.

**Fig. 5:**
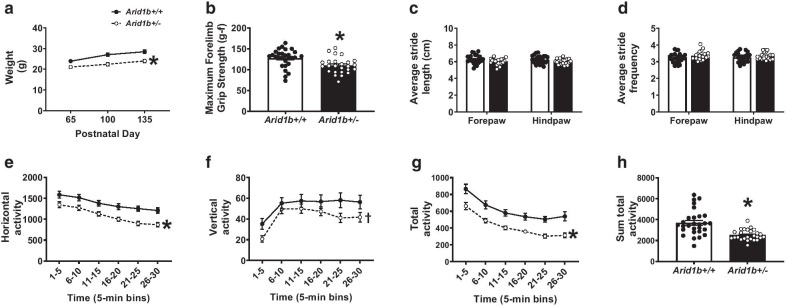
Adult *Arid1b*^+/−^ have diminished physical size, strength and motor ability. **a** Adult *Arid1b*^+/−^ weigh less compared to *Arid1b*^+/+^ adults and throughout behavioral testing. **b** In a forelimb grip strength assay, *Arid1b*^+/−^ showed decreased maximum forelimb force exerted, indicating reduced muscle strength. **c**, **d** Despite their smaller size, *Arid1b*^+/−^ showed normal stride length (**c**) and stride frequency (**d**) in fore and hind paws using DigiGait automated gait analysis, indicating normal ambulation and motor coordination of the limbs. **e**–**h** In open field exploration assay, horizontal, vertical and total activity were recorded for a 30-min period. Data are shown over time in 5-min bins. *Arid1b*^+/−^ were hypoactive with reduced horizontal activity (**e**), vertical activity (**f**) and total activity (**g**). **h** When summed over the 30-min session, *Arid1b*^+/−^ had less total activity compared to *Arid1b*^+/+^, indicating a robust motor deficit. For **a** and **b**, *Arid1b*^+/+^ *N* = 28, *Arid1b*^+/−^* N* = 25, for **c** and **d** *Arid1b*^+/+^ *N* = 21, *Arid1b*^+/−^ *N* = 19, for **e**–**h** *Arid1b*^+/+^ *N* = 28, *Arid1b*^+/−^* N* = 26. *p < .05 versus *Arid1b*^+/+^ by repeated measures two-way ANOVA or student’s unpaired *t* test. ^†^*p* < .09 versus *Arid1b*^+/+^ by repeated measures two-way ANOVA. Error bars represent mean ± SEM

**Fig. 6:**
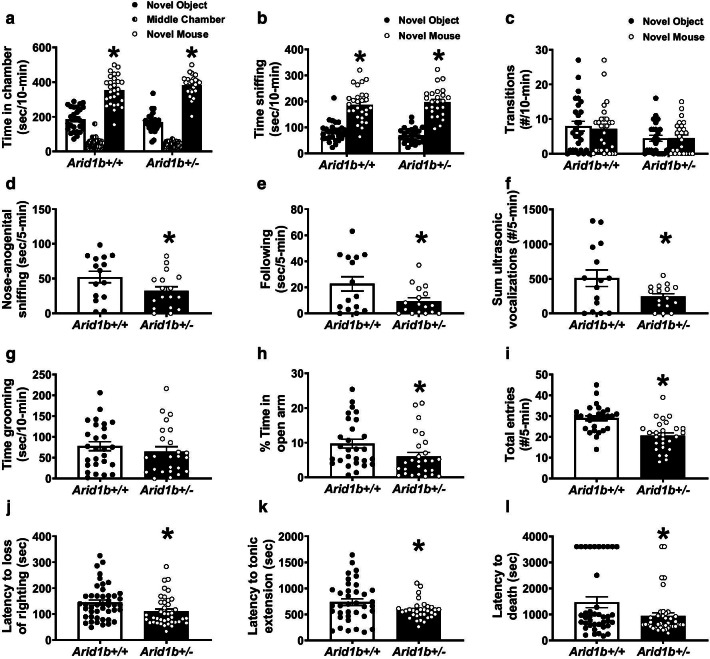
*Arid1b*^+/−^ exhibited mild ASD-relevant social communication phenotypes in the direct reciprocal social interaction and seizure susceptibility but no repetitive behavior. Three-chambered social approach is a standard measure of social behavior. It was used as a preliminary evaluation tool for sociability. While both genotypes exhibited normal social approach, i.e., they spent more time in the chamber with a novel mouse or time sniffing the novel mouse compared to a novel object), the *Arid1b*^+/−^ made fewer total entries during the task. **a** *Arid1b*^+/−^ showed no deficits compared to *Arid1b*^+/+^ littermates in 3-chambered social approach in time spent in chamber with the novel mouse, **b** time spent sniffing the novel mouse compared to novel object also indicated sociability in both genotypes. **c** *Arid1b*^+/−^ made fewer transitions compared to *Arid1b*^+/+^. **d**–**f** Next, we moved to a more sensitive task to observe social dyad interactions. During male–female social dyad interactions, males are introduced to a *Arid1b*^+/+^, novel estrous female for 5 min in a novel, clean environment and social investigative and situational behaviors are evaluated and male-emitted ultrasonic vocalizations (USVs) are counted. **d** Adult *Arid1b*^+/−^ males spent less time nose-to-anogenital sniffing and **e** less time in a following posture/behavior, the two main components of this interaction, indicating reduced social behavior in this task. **f** *Arid1b*^+/−^ males also emitted fewer USVs during the dyad interactions compared to *Arid1b*^+/+^ male littermates which also suggested impaired social communication. **g** No increased or decreased self-grooming was observed. **h**–**i** We used a gold standard assay of anxiety, the elevated plus maze, to observe anxiety-like behavior. **h** *Arid1b*^+/−^ spent fewer total seconds on the open arm which usually indicates anxiety-like behavior. **i** *Arid1b*^+/−^ also showed fewer total entries between arms, which suggests this task may be confounded by the data in Fig. 2, the robust motoric deficit. **j**–**l** With a high concordance of epilepsy in ASD, and excitatory inhibitory balance being a prominent theory, we investigated seizure susceptibility in *Arid1b*^+/−^. Mice are injected intraperitoneally with pentylenetetrazol (PTZ) chemoconvulsant, and latency to seizure events including loss of righting, tonic–clonic seizure and death were recorded. **j** *Arid1b*^+/−^ showed seizure vulnerability and susceptibility by decreased in time to loss of righting after PTZ administration (**k**) faster onset to tonic–clonic extension and **l** latency to death, compared to *Arid1b*^+/+^. For **a**–**c**, Arid1b^+/+^ *N* = 28, *Arid1b*^+/−^* N* = 25, for **d**–**f** *Arid1b*^+/+^ *N* = 15, *Arid1b*^+/−^* N* = 17, in **g**, *Arid1b*^+/+^ *N* = 28, *Arid1b*^+/−^* N* = 25, for **g** and **h**, *Arid1b*^+/+^ *N* = 28, *Arid1b*^+/−^* N* = 27, for **j**–**l**, *Arid1b*^+/+^ *N* = 45, *Arid1b*^+/−^* N* = 40. * *p* < .05 versus *Arid1b*^+/+^ by repeated-measures two-way ANOVA or Student’s unpaired *t* test. Error bars represent mean ± SEM

**Fig. 7:**
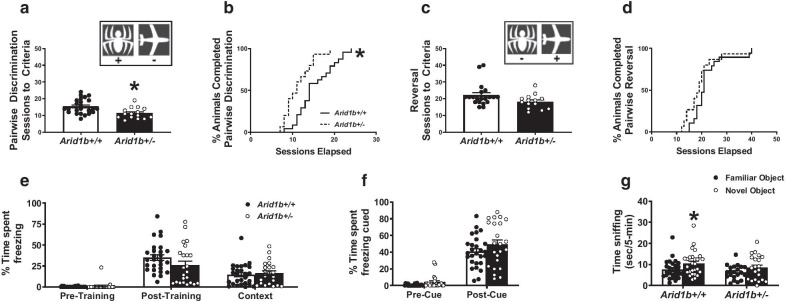
Largely intact learning and memory in *Arid1b*^+/−^. To assess learning and memory and behavioral flexibility in a computerized automated task, that does not rely heavily on motor skills, we utilized a visual pairwise discrimination touchscreen task. Mice are food-restricted and trained to initiate and execute trials by touching their noses to the touchscreen. Mice are then trained to associate an image (a spider or an airplane, A inset) with a food reward. **a** *Arid1b*^+/−^ required fewer sessions to reach criteria of 80% accuracy for two consecutive days compared to *Arid1b*^+/+^ littermates indicating no deficits in ability to acquire visual discrimination. **b** As a group, *Arid1b*^+/−^ completed acquisition of the pairwise discrimination task faster than *Arid1b*^+/+^, with larger proportions of mice finishing sooner (survival curve). Once animals achieved task criteria, they underwent reversal of the task where the opposite image now becomes the correct image (C, inset). **c** and **d** *Arid1b*^+/−^ showed no behavioral inflexibility or deficits in learning and memory in pairwise discrimination reversal compared to *Arid1b*^+/+^ by **c** no difference in number of sessions required to reach criteria (**d**) and no difference in proportion of mice reaching criteria of 75% accuracy for two consecutive days (survival curve, D). **e** and **f** In a canonical learning and memory tasks with less motor involvement, Pavlovian contextual and auditory cue fear conditioning, *Arid1b*^+/+^ and Arid1b^+/−^ demonstrated normal associative learning and memory abilities. Learning and memory was assessed by percentage of time spent freezing after associating a context and tone cue with a 0.5 mA foot shock. **e** *Arid1b*^+/−^ successfully acquired fear memory during training and showed intact contextual memory of their environment 24 h later. There was no difference in % freezing between *Arid1b*^+/+^ and *Arid1b*^+/^. **f** *Arid1b*^+/−^ also showed intact cued learning of an auditory tone compared to *Arid1b*^+/+^. Recognition memory was tested using a novel object recognition test in an open area. Mice are habituated to an open field arena for 24 h, familiarized for 10 min to two identical objects equally spaced in the arena. After an hour long intertrial interval, one object is replaced with a novel one and mice are allowed to explore for 5 min. Time spent sniffing the objects is recorded. Mice exhibit novel object recognition when they spend more time investigating the novel object compared to the familiar object. **g** *Arid1b*^+/+^ successfully showed a preference for the novel object and spent more time sniffing the novel object whereas *Arid1b*^+/−^ failed to show a preference and spent equal time sniffing both objects. For (**a** and **b**), *Arid1b*^+/+^ *N* = 24, *Arid1b*^+/−^* N* = 15, for (**c** and **d**), *Arid1b*^+/+^ *N* = 20, *Arid1b*^+/−^* N* = 14, for **e** and **f**, *Arid1b*^+/+^ *N* = 27, *Arid1b*^+/−^* N* = 23, for (**g**), *Arid1b*^+/+^ *N* = 27, *Arid1b*^+/−^* N* = 21. * *p* < .05 versus *Arid1b*^+/+^ by repeated measures Two-Way ANOVA or Student’s unpaired *t* test. Error bars represent mean ± SEM

**Fig. 8:**
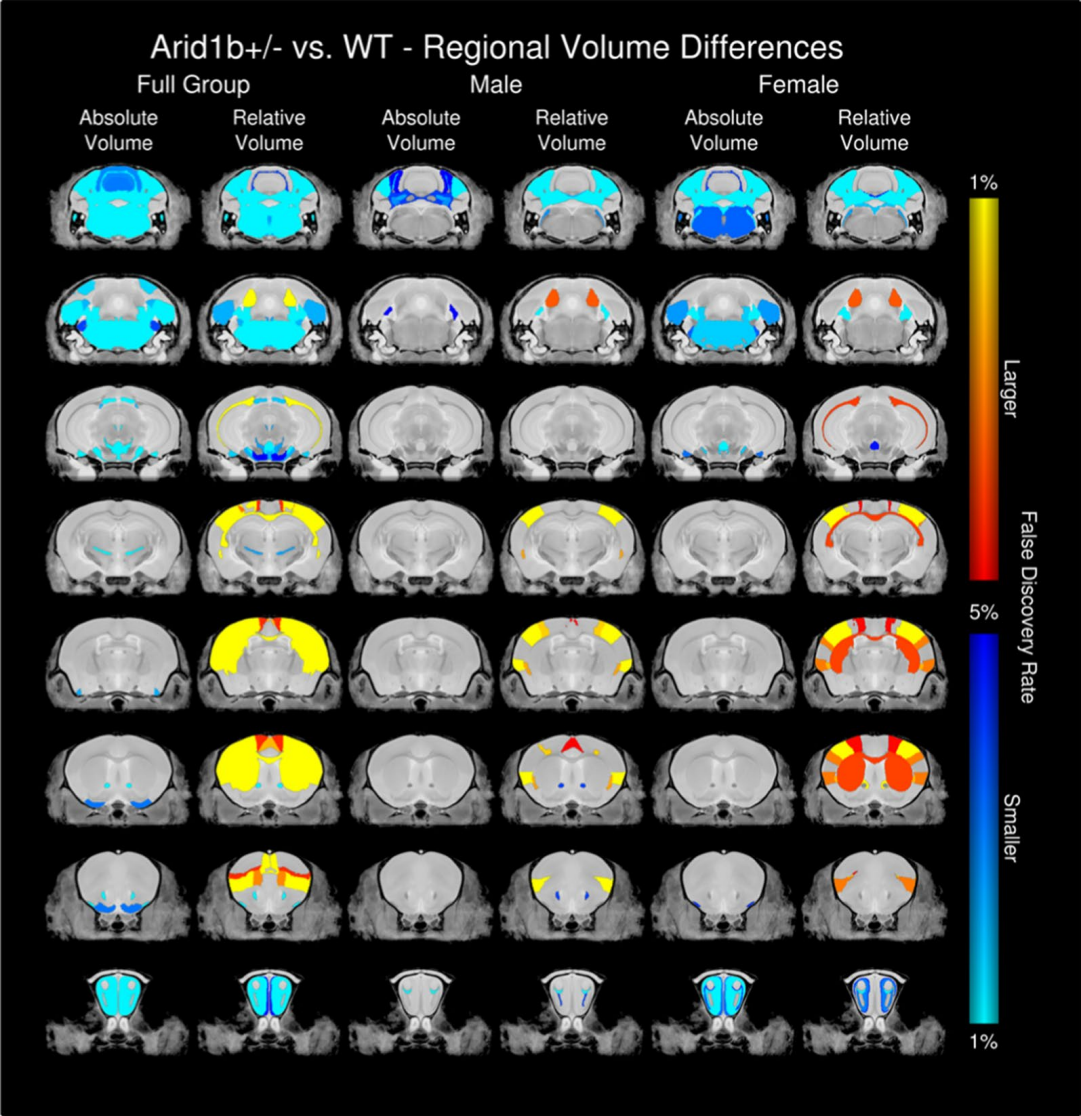
Structural Differences in Adult *Arid1b*^+/−^ Mice. Neuroanatomical difference found throughout the brain in the ex vivo adult brain cohort. Shown in both absolute and relative volume in the full group (including both sexes), males, and females. Absolute volume differences are measured in mm^3^, whereas relative volume differences are calculated from a linear model in which the total brain volume is used as a covariate

For the behavioral testing, Cohort 1 was sampled from 12 litters and was tested in elevated plus maze, light ↔ dark exploration, novel object recognition, social approach, male female social dyad interactions, self-groom, contextual and cued fear conditioning, hot plate, grip strength and lethal dose PTZ. Cohort 2 was sampled from 12 litters and was tested in pairwise discrimination and reversal touchscreen task followed by a lethal dose of PTZ. All male and female mice were used from every litter. In cohort 1, the order of testing was as follows with at least 48-h separating tasks: (1) PND3, 5, 7, 9 ultrasonic vocalizations or PND2, 4, 6, 8, 10, 12, 14 developmental milestones, (2) elevated plus-maze at 10 weeks of age, (3) light ↔ dark exploration task at 10 weeks of age, (4) open field locomotion at 11 weeks of age, (5) balance beam walking at 11 weeks of age, (6) spontaneous alternation at 12 weeks of age, (7) rotarod at 12 weeks of age, (8) novel object recognition at 13 weeks of age, (9) self-groom at 15 weeks of age, (10) social approach at 15 weeks of age, (11) male female social dyad interaction at 16 weeks of age, (12) grip strength at 17 weeks of age, (13) hot plate at 17 weeks of age, (14) fear conditioning at 18 weeks of age, and (15) lethal dose chemoconvulsant at 19 weeks of age. In cohort 2, ultrasonic vocalizations or developmental milestones were conducted between PND2 and 14 in separate litters, weight restriction for touchscreen assay began at 7 weeks of age, pretraining began at 9 weeks of age and touchscreen testing continued daily until completion, DigiGait was conducted at 17 weeks of age and lethal dose chemoconvulsant at 18 weeks of age. All behavioral assays in all cohorts were performed between 9 am–4 pm PST (ZT2-ZT9). For Fig. 5a–c, *Arid1b*^+/+^ *N* = 40, *Arid1b*^+/−^* N* = 19, for (d–i), *Arid1b*^+/+^ *N* = 18, *Arid1b*^+/−^* N* = 29. For Fig. 6a, b, *Arid1b*^+/+^ *N* = 28, *Arid1b*^+/−^* N* = 25, for (c–d) *Arid1b*^+/+^ *N* = 21, *Arid1b*^+/+^ *N* = 19, for (e–h) *Arid1b*^+/+^ *N* = 28, *Arid1b*^+/−^* N* = 26. ^†^*p* < 0.09 versus *Arid1b*^+/+^ by repeated measures two-way ANOVA. For Fig. 7 a–c, Arid1b^+/+^ *N* = 28, *Arid1b*^+/−^* N* = 25, for (d–f) *Arid1b*^+/+^ *N* = 15, *Arid1b*^+/−^* N* = 17, in (g), *Arid1b*^+/+^ *N* = 28, *Arid1b*^+/−^* N* = 25, for (g–h), *Arid1b*^+/+^ *N* = 28, *Arid1b*^+/−^* N* = 27, for (j–l), *Arid1b*^+/+^ *N* = 45, *Arid1b*^+/−^* N* = 40. For Fig. 8a, b, *Arid1b*^+/+^ *N* = 24, *Arid1b*^+/−^* N* = 15, for (c–d), *Arid1b*^+/+^ *N* = 20, *Arid1b*^+/−^* N* = 14, for (e–f), *Arid1b*^+/+^ *N* = 27, *Arid1b*^+/−^* N* = 23, for (g), *Arid1b*^+/+^ *N* = 27, *Arid1b*^+/−^* N* = 21. For Figs. 5, 6, 7, and 8 significance was measured by repeated measures two-way ANOVA or student’s unpaired *t* test.

## Results

### Validation of reduced *Arid1b* and broad differential expression signatures in adult cerebellum

Quantitative reverse-transcription polymerase chain reaction (qRT-PCR) and Western blot were used to confirm that adult *Arid1b*^+/−^ mice of both sexes had reduced mRNA and protein levels in cortex and cerebellum samples compared to *Arid1b*^+/+^ mice (Fig. 1a–c). As expected, *Arid1b*^+/−^ did indeed show 50% relative *Arid1b* transcript expression via qRT-PCR in both cortex and cerebellum lysates, compared to *Arid1b*^+/+^ (Fig. 1a, p < 0.05 for cortex and cerebellum). Moreover, this translated to nearly half of the ARID1B protein level in *Arid1b*^+/−^ mice, compared to *Arid1b*^+/+^ mice in both structures analyzed (Fig. 1b, c, p < 0.05 for both cortex and cerebellum). Additionally, we performed exploratory transcriptomic analyses (RNA-seq) on cerebellum tissue from adult *Arid1b*^+/−^ and *Arid1b*^+/+^ that underwent behavioral experiments (one male and one female per genotype). Among 8942 genes that were robustly expressed, 373 genes were differentially expressed (DE) at FDR < 0.2 (*p* < 0.0083; 237 up and 136 down) and 72 DE at FDR of 0.05 (*p* < 0.0004; 21 up, 51 down) (Fig. 1a, Additional file 1: Table S1). *Arid1b* was differentially expressed (log2 FC =  −0.37, *p* = 0.0037, FDR = 0.14; Fig. 1d). Gene set enrichment analysis of Gene Ontology (GO) terms identified axon and synaptic terms among upregulated genes and transcriptional regulation and RNA processing enriched among downregulated genes (Fig. 1e). We found differential expression of autism-relevant genes in our DE gene list that are also associated with cell adhesion (*Dscam*, *Reln*) or gene regulation (*Kat2b*, *Kmt2c*, *Med13*/*Med13l*, *Tbl1xr1*, *Ubn2*). These analyses validated *Arid1b* haploinsufficiency at the mRNA and protein level and are suggestive of broad transcriptional pathology in adult cerebellum.

### Hydrocephaly

There was an increased incidence of hydrocephaly in the *Arid1b*^+/−^ mice compared to other mice in both the Sacramento and Toronto colonies. Prior to the beginning of this study, 667 *Arid1b*^+/−^ mice were born in the Toronto facility, of which 3.1% (21) had hydrocephaly, compared to 0.2–0.3% in other mice on the same C57BL/6NCrl background; these mice were humanely euthanized or removed from the analysis. Of the 89 *Arid1b*^+/−^ mice born in the Sacramento laboratory, 55 were used and 12.7% (7) were excluded or died due to hydrocephalus (equal rates between sexes). Of the 82 *Arid1b*^+/+^ used in this study, only one was excluded due to hydrocephalus (1.2%). Hydrocephalus has been previously reported in *Arid1b*^+/−^ mice models at rates of 5.5% to 6.6% [19, 21]. The rates in this study were based on mice that had severe hydrocephaly and thus were excluded from the behavioural outcomes; however, as described in more detail below, it should be noted that the ventricular system as a whole was enlarged in *Arid1b*^+/−^ mice throughout development (*q* = 0.005), particularly the lateral ventricles (*q* = 2.0 × 10^−12^).

### Developmental behavioral findings

*Arid1b*^+/−^ pups were impaired on several parameters of developmental milestones including growth, reflexes, and pup ultrasonic vocalization (USVs) emissions. It should be noted that no sex differences were found behaviorally throughout development and thus the sexes were pooled. Figure 2a illustrates isolation induced USVs over a developmental time course. A significant effect of age was detected on the rate of calls over time (*F*_(3, 171)_ = 6.68, *p* ≤ 0.005, two-way repeated measures ANOVA). Genotype effects across time were also detected (*F*_(1, 57)_ = 6.67, *p* < 0.02, one-way repeated measures ANOVA), prominently at PND3 and 5 (*Arid1b*^+/−^ vs. *Arid1b*^+/+^, Bonferroni–Sidak post hoc, Additional file 2: Table S2) where *Arid1b*^+/−^ produced fewer calls each day and showed a delayed shift in day of peak calls compared to *Arid1b*^+/+^. Sum of calls collected were fewer in the *Arid1b*^+/−^ (Fig. 2b; *t*_(58)_ = 3.08, *p* ≤ 0.005, student’s *t* test), compared to *Arid1b*^+/+^ littermates. Core temperature (Fig. 2c; *F*_(1, 57)_ = 3.92, NS) was not different by genotype, a variable known to alter pup USVs [60].

Growth, determined by body weight, length, and head width, was delayed and stunted as *Arid1b*^+/−^ were smaller in these metrics (Fig. 2d; *F*_(1, 45)_ = 14.8, *p* ≤ 0.005; Fig. 2e; *F*_(1, 45)_ = 14.07, *p* ≤ 0.005; Fig. 2f; *F*_(1, 45)_ = 8.10, *p* ≤ 0.01, respectively, two-way repeated measures ANOVA), indicating atypical growth and development. Post hoc analysis revealed *Arid1b*^+/−^ weighed less on PND6-12, were smaller on PND10-12, and had narrower heads on PND12 versus *Arid1b*^+/+^ (Sidak post hoc for all analysis).

*Arid1b*^+/−^ did not demonstrate hypotonia and physical core coordination deficits as they did not have longer latencies to right their bodies dorsally, but, longer latencies to reverse from an inclined position in negative geotaxis and time to traverse out of circle were detected in *Arid1b*^+/−^, respectively (Fig. 2g; *F*_(1, 45)_ = 3.71, *p* = 0.06; Fig. 2h; *F*_(1, 45)_ = 4.54, *p* ≤ 0.05; Fig. 2i; *F*_(1, 45)_ = 4.34, *p* ≤ 0.05, two-way repeated-measures ANOVA, Additional files 2–4: S2–S4), indicate delayed motoric reflexes, complex limb coordination, and onset of walking skills. Clear divergence in physical metrics were detected in mid and late development. As the pups developed, some metrics were difficult to collect with reliable accuracy at every time point, which may contribute to the incomplete penetrance of the phenotypes. However, the strong genotype effect was convincing that *Arid1b*^+/−^ diverged by numerous major developmental metrics.

### Developmental structural findings

The results of the linear mixed effects model showed that *Arid1b*^+/−^ (genotype) had a large effect on several brain structures (Fig. 3, Additional file 5: Table S5). Across ages, there was an overall loss in total brain volume (*q* = 0.02). The strongest differences were seen in the cerebellum and hippocampus. The cerebellum as a whole was smaller in the *Arid1b*^+/−^ mice (*q* = 3 × 10^−8^); this affected all areas including the vermal regions (*q* = 8 × 10^−7^), the hemispheric regions (*q* = 0.0004) with strong effects in the arbor vitae (*q* = 3 × 10^−11^), and the deep cerebellar nuclei (*q* = 2 × 10^−9^). Notable differences were found in the hippocampal regions, including an increase in size of the CA1 (*q* = 0.003), CA2 (*q* = 0.001), CA3 (*q* = 2 × 10^−8^), and the dentate gyrus (*q* = 6 × 10^−7^).

We found a significant interaction between age and genotype, indicating that *Arid1b*^+/−^ mutants demonstrate different growth rates, mainly an increased growth rate in the hippocampus (Fig. 3b, q = 0.00008) and a decreased growth rate in the cerebellum (Fig. 3c, q = 0.00004) compared to *Arid1b*^+/+^. Differences in growth rate were seen in all cerebellar areas including the vermal regions (*q* = 0.0006), hemispheric regions (*q* = 0.03), the deep cerebellar nuclei (Fig. 3d, q = 0.0001), and the arbor vitae (*q* = 4 × 10^−9^) (Fig. 3). Cerebellar growth rates corresponded to differences in adulthood, indicating that all areas of the cerebellum grew at a slower rate, thereby resulting in the adult differences. In addition to the cerebellar white matter, there were strong differences in white matter fiber tracts in general (*q* = 0.00009); however, the growth rate was increased in the *Arid1b*^+/−^ mutants.

We also observed a significant interaction between sex and genotype, and additionally several differences were found in the three-way interaction between age, sex, and genotype (Fig. 3a). Figure 4a highlights the top 50 structures (based on percent difference) for both larger and smaller regions in the *Arid1b*^+/−^ mutants versus the *Arid1b*^+/+^ mice. Regardless of whether the difference was larger or smaller at PND60, affected structures diverged earlier in the males, with differences seen as early as PND7, while in females, the differences did not emerge until after PND40 (Fig. 4b, c), thus displaying a sex differences across development.

### Adult behavioral findings

Similar to the developmental outcomes, there were no sex differences found in the adult behavioral tasks; and therefore, the sexes were pooled for analysis. Adult *Arid1b*^+/−^ remained smaller and weaker in measurements of size and strength. Figure 5a illustrates adult *Arid1b*^+/−^ weighed less compared to *Arid1b*^+/+^. Body weight was collected across the study and all animals gained mass across all time points PND65, PND100 and PND135 (Fig. 5a; *F*_(2, 102)_ = 92.53, *p* ≤ 0.005, two-way repeated measures ANOVA) but less by the *Arid1b*^+/−^ mice (Fig. 5a; *F*_(1, 51)_ = 15.22, *p* ≤ 0.005, two-way repeated measures ANOVA), compared to *Arid1b*^+/+^ sex-matched littermates. *Arid1b*^+/−^ showed decreased maximum forelimb force exerted, indicating reduced muscle strength by a forelimb grip strength assay (Fig. 6b; *t*_(51)_ = 3.08, *p* ≤ 0.005, student’s unpaired *t* test). Nuanced motoric behaviors, such as gait, balance, posture, stride, and motor coordination can be measured using innovative automated treadmill systems, DigiGait™. Despite their smaller size, *Arid1b*^+/−^ did not show differences in stride length (Fig. 5c; *F*_(1, 42)_ = 3.09, NS, two-way repeated measures ANOVA) nor stride frequency (Fig. 5d; *F*_(1, 43)_ = 2.59, NS, two-way repeated measures ANOVA) in either fore or hind limbs. While a decrease in stride length and an increase in stride frequency were expected due to their diminished size, the lack of deficit in these two major parameters indicate intact motor coordination of the limbs.

Adult *Arid1b*^+/−^ were impaired on multiple parameters of gross motor skills using an open field novel arena assay of locomotion. *Arid1b*^+/−^ were hypoactive over a 30-min session. Differences for horizontal and vertical activity and total distance traversed in the novel open field, were significant, representing normal habituation to the novelty of the open field in both genotypes (Fig. 5e–h; horizontal *F*_(5, 260)_ = 42.02, *p* ≤ 0.005; vertical *F*_(5, 260)_ = 26.06, *p* ≤ 0.005; total activity *F*_(5, 260)_ = 52.2, *p* ≤ 0.005, two-way repeated measures ANOVA). *Arid1b*^+/−^ mice were hypoactive by horizontal and vertical activity during all 5 min binned sessions (Fig. 5e; *F*_(1, 52)_ = 14.71, *p* ≤ 0.005; Fig. 5f; *F*_(1, 52)_ = 2.80, *p* ≤ 0.005, two-way repeated measures ANOVA), by total distance traversed over time (Fig. 5g; *F*_(1, 52)_ = 20.96, *p* ≤ 0.005, two-way repeated measures ANOVA) and summed across the 30-min session (Fig. 5h; *t*_(52)_ = 4.58, *p* ≤ 0.005), indicating a clear, corroborated motor deficit.

Behaviors relevant to ASD were assessed using two corroborating assays of social behavior, as described previously [12, 50–52]. Sociability scores from the automated three-chambered social approach task were typical and showed significant sociability (Fig. 6a; two-way repeated measures ANOVA main effect of genotype: *F*_(1, 51)_ = 3.016, *p* > 0.05; post-hoc novel object vs. novel mouse: *Arid1b*^+/+^ *p* < 0.001, *Arid1b*^+/−^* p* < 0.001). Both *Arid1b*^+/−^ and *Arid1b*^+/+^ mice exhibited significantly more time social sniffing, which was defined as time spent within 2 cm of the wire cup, with the head facing the wire cup containing the stimulus mouse, as compared to the time spent sniffing the novel object, using the same body point detection settings (Fig. 6b; two-way repeated measures ANOVA, main effect of genotype: *F*_(1, 51)_ = 0.03, *p* < 0.005; *Arid1b*^+/+^ *p* < 0.001, *Arid1b*^+/−^* p* < 0.001). *Arid1b*^+/−^ mice showed fewer entries into the side chambers (Fig. 7c; two-way repeated measures ANOVA, main effect of genotype: *F*_(1, 51)_ = 8.49, *p* = 0.05), indicating that reduced ARID1B resulted in less general exploratory activity throughout the 3-chambered apparatus during the social approach assay. However, it did not adversely affect the outcome as both genotypes illustrated intact sociability.

Social deficits were observed on investigative and social parameters in male *Arid1b*^+/−^ mice when compared to littermate *Arid1b*^+/+^ during the male–female reciprocal dyad social interaction test. Figure 6d–f illustrates male–female social interaction parameters during a session of reciprocal interactions between subject male *Arid1b*^+/−^ and *Arid1b*^+/+^ mice paired with an unfamiliar estrous BL/6NJ female. *Arid1b*^+/−^ exhibited less time in nose-to-anogenital sniffing (Fig. 6d; *t*_(30)_ = 1.97, *p* ≤ 0.05, Student’s unpaired *t* test) and following behavior (Fig. 6e; *t*_(30)_ = 2.27, *p* ≤ 0.05, Student’s unpaired *t* test). Other parameters collected during the social interaction such as nose to nose sniffing, exploration, and grooming were not significant. Once again, as adults, ultrasonic call emissions were fewer in the *Arid1b*^+/−^ (Fig. 6f; *t*_(29)_ = 2.12, *p* ≤ 0.005, Student’s unpaired *t* test), which may be attributed to reduced social communication but could also be the result of being smaller, with less pronounced larynx musculature [60–62].

Self-grooming is a useful measure of repetitive behavior in models of NDDs with high repetitive behaviors. No difference was observed on repetitive self-grooming behavior (Fig. 6g; *t*_(50)_ = 0.82, NS, Student’s unpaired *t* test). No other reported repetitive behaviors, such as circling, jumping or back flipping, were observed during this empty cage observational assay.

Anxiety-like behavior was assessed in the elevated plus-maze. *Arid1b*^+/−^ mice spent less time on the open arm and had fewer total entries calculated by the open and closed arm entries summed (Fig. 6h; *t*_(53)_ = 2.10, *p* ≤ 0.05 and Fig. 6i; *t*_(53)_ = 4.61, *p* ≤ 0.001, Student’s unpaired *t* test). This is consistent with less motoric activity in the *Arid1b*^+/−^ mice and confounds a clear interpretation of anxiety-like behavior, although anxiety metrics indicate this to be a phenotype. Moreover, *Arid1b*^+/−^ mice were not significantly different from *Arid1b*^+/+^ controls in the light/dark conflict task as both groups spent the similar amounts of time in the dark chamber (*p* < 0.05), had the same latency to the first transition (*p* < 0.05), and made similar transitions (*p* < 0.05), not demonstrating a clear anxiety-like phenotype.

Seizures were provoked using pentylenetetrazol (PTZ; 80 mg/kg, i.p.) in *Arid1b*^+/−^ and *Arid1b*^+/+^ mice littermates, as previously described [59]. Latencies to generalized clonic seizure (loss of righting reflex), tonic extension, and death were collected as a preliminary characterization of seizure, subthreshold epileptiform activity, and imbalances in the excitatory/inhibitory homeostasis. Pentylenetetrazol (PTZ) is a non-competitive GABA_A_ antagonist leading to hyperexcitability [63, 64]. *Arid1b*^+/−^ mice exhibited faster loss of righting reflexes (Fig. 6j; *t*_(83)_ = 2.49, *p* ≤ 0.02, Student’s unpaired *t* test), tonic hindlimb extension (Fig. 6k; *t*_(67)_ = 2.11, *p* ≤ 0.04, Student’s unpaired *t* test), and death (Fig. 6l; *t*_(77)_ = 2.56, *p* ≤ 0.02, Student’s unpaired *t* test).

Learning and memory was evaluated via fear conditioning using two components, a 24-h contextual and a 48-h cued fear conditioning. High levels of freezing were observed, subsequent to the conditioned stimulus–unconditioned stimulus pairings, on the training day, across genotypes (Fig. 7e; training *F*_(1, 48)_ = 108.9, *p* ≤ 0.001; genotype training *F*_(1, 48)_ = 1.74, NS, two way repeated measures ANOVA). No significant effects of genotype on freezing levels were observed 24-h later during the contextual phase (Fig. 7e; *t*_(49)_ = 0.21, NS, Student’s unpaired *t* test). High levels of freezing were observed, after the auditory cue stimulus was introduced, on cued conditioning day, across genotypes indicating no impairments in cued conditioning (Fig. 7f; effect of cue *F*_(1, 48)_ = 205.4, *p* ≤ 0.001; genotype *F*_(1, 48)_ = 2.86, NS, two-way repeated measures ANOVA); the same was observed 24-h later during the contextual phase (Fig. 7e; *t*_(49)_ = 0.21, NS, Student’s unpaired *t* test).

Cognitive abilities as measured by the novel object recognition task were tested. All automated scores collected via Ethovision were manually confirmed by a trained blinded observer [43]. *Arid1b*^+/−^ mice did not exhibit typical novel object preference (Fig. 7g; *Arid1b*^+/+^, *p* < 0.01 and *Arid1b*^+/−^* p* > 0.05). Both genotypes explored the two identical objects similarly during the automated familiarization phase (data not shown).

We utilized innovative computerized touchscreen assays with high translational relevance to evaluate visual discrimination and behavioral flexibility attributable to both hippocampal and cortical circuitry [54]. Figure 7a–c illustrate *Arid1b*^+/−^ mice required significantly fewer sessions to a stringent criterion of completing at least 30 trials, at an accuracy of 80% or higher, on two consecutive days, when compared to littermate controls. *Arid1b*^+/−^ mice needed fewer sessions over the training days to learn to discriminate between two images displayed on the touchscreen (Fig. 7a; *t*_(37)_ = 3.07, *p* ≤ 0.004, Student’s unpaired *t* test). Analysis of survival curves, i.e., percentage of mice that reached the 80% accuracy criterion on each training day, indicated that the percentage of mice that reached this criterion was significantly higher in *Arid1b*^+/−^ (Fig. 7b, X = 8.88, *p* ≤ 0.003, Gehan Breslow Wilcoxon Chi Square test). During reversal, designation of the correct and incorrect stimulus was switched. No genotype effect was observed in the second, more difficult, reversal phase as shown by sessions to reversal criteria (Fig. 7c; *t*_(32)_ = 1.92, NS, Student’s unpaired *t* test) and survival curves of mice reaching criterion over time (Fig. 7d, *X* = 2.57, NS, Gehan Breslow Wilcoxon Chi-Square test).

### Adult structural findings

Total brain volume of the *Arid1b*^+/−^ mutants was 2.21% smaller than their corresponding *Arid1b*^+/+^ littermates. However, this did not reach significance (*p* = 0.02, False Discovery Rate (FDR) *q* = 0.37, 420 ± 14 mm^3^ for *Arid1b*^+/−^; 429 ± 12 mm^3^ for *Arid1b*^+/+^, Fig. 8, Additional file 6: Table S6). In the 282 regions examined, 79 were significantly different at *q* < 0.05. The main olfactory bulb was significantly smaller (− 6.10%, *q* = 0.0007), with its olfactory subregions ranging from − 5.96% to − 10.41% (Fig. 8). The brainstem was also significantly smaller (− 3.13%, *q* = 0.046), due to a smaller hindbrain and pons (− 5.53%, *q* = 0.0004 and − 4.99%, *q* = 0.0005, respectively). Overall, white matter was significantly smaller (− 3.71%, *q* = 0.046). However, this difference stemmed from cerebellar and cranial nerve fibers, and not from the largest white matter tracts in the mouse brain, the fimbria and corpus callosum, commonly found altered in models of NDDs (Fig. 8). The largest differences were in the cerebellum (− 6.77%, *q* = 0.00007), with the cerebellar cortex decreasing by − 6.65% (*q* = 0.0001) and the deep cerebellar nuclei (DCN) decreasing by − 10.99% (*q* = 4 × 10^−8^). Relative volume differences were also measured by covarying for total brain volume. The majority of differences found in relative volume were consistent with absolute volume (Fig. 8). However, there were significant increases in the relative volume of the cortex (*q* = 0.01), the striatum (*q* = 0.006), and the corpus callosum in the *Arid1b*^+/−^ (*q* = 0.007).

Figure 8 shows a visual representation of the differences found throughout the brain in the *Arid1b*^+/−^ mutants. The *Arid1b*^+/−^ mutation seemed to have a more widespread effect on females: 47 of the 282 regions were significantly different, while only 9 were different in the males (Fig. 8). However, there was no sex by genotype interaction found in the adult mice, indicating that the mutation had similar effects in both sexes.

## Discussion

While *Arid1b*^+/−^ mice have been previously examined [19–21], we present an in-depth characterization of behavior and whole brain structure through development in a newly generated model. We show dramatic growth and motor deficits, confirm previous anxiety and social behavioral phenotypes, and highlight several novel and contradictory neuroanatomical differences that encompass the cerebellum, hippocampus and corpus callosum. For example, the corpus callosum is larger in our *Arid1b*^+/−^ mice throughout development which contrasts with both previous work and a subset of the human cases. We also found a novel sex dependence in our neuroanatomical findings; the differences in males have an earlier emergence, but the male and female phenotype converge by adulthood.

We first validated reduced *Arid1b* expression in this novel mouse line. As expected, *Arid1b*^+/−^ mice exhibited reduced mRNA and protein levels in adult cortex and cerebellum. While the focus of this study was behavior and brain structure, we performed exploratory transcriptomic analysis in adult cerebellum from mice that underwent behavioral analyses. This limited analysis was not sufficiently powered to detect subtle changes or differences between sexes, nonetheless we found down-regulation of transcriptional and RNA processing regulators and up-regulation of axonal and synaptic genes. Previous RNA-seq studies of *Arid1b*^+/−^ mice have not examined cerebellum, and our results are suggestive of transcriptional pathology associated with behavioral and anatomical phenotypes. The specific genomic ARID1B binding targets and their sensitivity to *Arid1b* haploinsufficiency remain to be defined in the brain, and it will be critical to understand the direct regulatory functions of ARID1B to understand the root molecular mechanisms linked to *Arid1b* haploinsufficiency. Given the exploratory nature of this analysis, future work will be required to robustly capture transcriptional changes in *Arid1b*^+/−^ cerebellum, validate the impact on protein expression, and link molecular to behavioral and anatomical phenotypes.

While there are some mixed results within previous behavioral investigations of *Arid1b*^+/−^ mice, there is a general consensus in three areas [19–21]. (1) A generalized increase in anxiety-like levels; corroborated in our findings. (2) A generalized decrease in sociability. We observed normal 3-chambered social approach, but found that male *Arid1b*^+/−^ mice spent less time engaging in social interactions in the male–female social dyad interaction assay [50, 51]. And finally, (3) an increase in repetitive behavior in *Arid1b*^+/−^ mice; our tests in self-grooming (Fig. 6g) and marble burying (data not shown) were unable to corroborate this phenotype in our line.

The notable developmental delay in the *Arid1b*^+/−^ mice, with substantial reductions in both size and basal locomotor activity, can cause interpretive issues. Control testing in open fields are commonly used in NDD. However, it cannot be overstated that the results of those observations may be driven by size or low motor skills [46, 47]: a small mouse tested in large chambers will always be at a disadvantage. We had the unique opportunity to observe the impact of an ~ 15% size reduction and reduced locomotive activity on the control tasks and sophisticated behavioral outcomes. While *Arid1b*^+/−^ are small and weak throughout life, they have preserved motor coordination, gait abilities, and motor learning (Fig. 6). Therefore, since during our comprehensive collection and discussion of developmental milestones, *Arid1b*^+/−^ did not demonstrate hypotonia and physical core coordination deficits; we do not believe the reduction in pup USV was confounded. Body weight and temperature were collected during pup call collection to assure that the reduced USV were not the result of being physically smaller as body weight is known to alter pup USV emission. Our laboratory has reported on this confound in numerous publications [11, 12] although this interpretation is unlikely, in our opinion, in this line.

*Arid1b*^+/−^ mice showed a deficit in novel object recognition, which aligns with cognitive deficits previously found [20]. However, when tested in motor-independent tasks, we did not observe this phenotype. *Arid1b*^+/−^ mice showed normal contextual and cued memory in a fear conditioning paradigm and showed normal or even slightly better performance in a pairwise discrimination task. By investigating learning and memory abilities that do not rely on high levels of movement and exploration, we avoided potential confounds regarding cognitive deficits. *Arid1b*^+/−^ mice were not impaired on motor-independent cognitive tasks. This was surprising as *ARID1B* is a common gene mutated in patients with intellectual disability, and learning difficulties are common in children with CSS and ASD [4, 11, 65]. This may reflect limitations with mice as a model system, or the high heterogeneity observed in human population. Previous groups reported learning and memory deficits without factoring in the motor impairment despite its phenotypic presence in these reports [46, 66]. This comprehensive profile of behavioral phenotypes is essential for bridging the gap and improving translational predictability.

Behavioral seizure susceptibility has not previously been investigated, but is relevant in both CSS and ASD [7]. We observed a strong seizure susceptibility phenotype in *Arid1b*^+/−^ mice (Fig. 6). *Arid1b*^+/−^ mice were functionally susceptible to PTZ-induced seizures, related to GABAergic neuron plasticity, we attribute this to an excitatory-inhibition imbalance, as with other genetic models of NDD [67–69]. This hypothesis corroborates histological analysis by Jung et al. [20].

As the behavior and imaging experiments were not performed on the same animals, we cannot draw direct links between the imaging and the behavior. However, we can speculate on possible connections between the findings. For example, the motor deficits found in these mice could be linked to both the cerebellar deficit that is seen throughout development and in the adult animals and the differences found in the primary motor cortex throughout development. Additionally, while we did not find any difference in repetitive behaviors or learning deficits in this specific cohort of mice, the previous studies on *Arid1b*^+/−^ mice did find increased repetitive behaviors. The striatum is often linked with increases in repetitive behavior; it is noteworthy that we found a relative volume increase in the size of the cerebellum in the *Arid1b*^+/−^ mice and this could be related to an increase in repetitive behaviors. Although, this also highlights the benefits of imaging and behavior performed on the same set of animals to allow for direct links [47].

In the original human CSS case studies, postmortem brain samples showed a thinning, or absence, of the corpus callosum. Recent mouse studies, therefore, focused on that region. Celen et al. reported a smaller corpus callosum in adult *Arid1b*^+/−^ mice [19], confirming what was seen in a subset human patients. Shibutani et al., report no histological abnormalities in the *Arid1b*^+/−^ mice [21]. In our developmental whole brain analysis, we found an increase in the corpus callosum size across development and no differences in adulthood. We also found an age by genotype interaction in the corpus callosum, indicating an increased growth rate. Another regional difference reported by Celen et al. was a decreased dentate gyrus. Again, contradicting this work, we found an increase in the size of the hippocampal formation across development. These inconsistencies with previous work could be attributable to multiple factors including background strain effects, as our mice were on a C57BL/6N background compared to the other studies (C57BL/6J), as well as interactions between age, sex, and genotype.

The original CSS case studies reported cerebellar and brain stem differences in patients [10], and these areas were drastically affected in our study (Additional file 5: Table S5). This confirms in the mouse what has only been reported in humans. Additionally, those case studies also report a Dandy-Walker malformation (hypoplasia of the cerebellar vermis with cystic dilation of the fourth ventricle), which is also seen in our mice. Both *ARID1B* and cerebellar abnormalities have been linked to ASD [70, 71]. In fact, in humans, one of the original MRI findings was an abnormal cerebellar vermis [71], and in mice, the cerebellum has been consistently linked to autism relevant genetic mutations [13, 14, 72–74]. The neuroanatomical phenotype of the *Arid1b*^+/−^ mouse is a smaller cerebellum, particularly of the white matter and vermal structures, in conjunction with a larger relative isocortex, hippocampus, and corpus callosum. This is consistent with several previously published mouse models related to autism, including *Chd8*^+/−^ [12, 75, 76], 22q11.2 [77], *Fmr1* [72], *Itgb3* [73], *En2* [13], *Nrxn1a* [13], and *Shank3* [13]. The relationship to* Chd8*^+/−^ mouse models would be expected as both models are related to chromatin modification. This suggests that a smaller cerebellum, with a larger cortex and corpus callosum, may delineate a neuroanatomical subset of ASD related to chromatin modification.

The neuroanatomical delay, interestingly, is sex dependent. Neuroanatomical differences in *Arid1b*^+/−^ emerge at PND7 in males, but PND40 in females (Fig. 4). This may explain some discrepancies with other preclinical single sex *Arid1b*^+/−^ papers. Previous authors did not look at sex differences outside of a few measures, and either used mixed groups [20], males [21], or females [19]. Interestingly, the genotype-based sex differences seen in the *Arid1b* mice have a similar effect as normal wild-type neuroanatomical sex differences [28]. Brain regions that are larger in adult male mice emerge earlier than those that are larger in female mice [28]. In that study the authors found several clusters of brain regions which had developmental growth trajectories that differed between male and female mice. One of those regions was the cerebellum, which was predominantly featured in our findings here. In that study the cerebellum showed little sex differences early in life but across puberty a sex difference emerged where the cerebellum became larger in females [28]. While it is just speculation, it is possible that the genotype effect emerges later in the cerebellum in females due to the increased growth in the cerebellum that is happening during that time period. Regardless, this data indicates that both sex and the *Arid1b*^+/−^ mutation contribute to a differential growth patterns between males and females and indicate caution in interpreting data from a single timepoint in a single sex.

### Limitations

There are a few limitations to this study. The first of which is that the imaging and behavioral testing were performed on different cohorts. This does not allow the ability to link the behavioral findings directly to specific neuroanatomy nor the reverse of linking the imaging findings to a specific behavioral trait. Second, the transcriptomic analysis study presented here was exploratory and needs replication at the protein level. Third, as attention deficits are often comorbid with NDD latency to respond correctly in the discrimination task could be a measure of attention; however, investigating attention in this manner was beyond the scope of this work, but it would be worth investigating in the future. Finally, this is one of several papers that have now examined *Arid1b*^+/−^ mice, and some of our findings are confirmatory, but also several are contradictory to previously published works in mouse and human subjects. However, we feel that both the behavior and imaging performed in this work are robust and reproducible, such that we have full confidence in the data presented herein.

## Conclusions

Overall, our behavioral findings are similar to earlier reports, although we observed a more subtle effect, with fewer findings in the ASD-relevant behaviors. Methodological differences, including test order, could account for our inability to see differences as robust as the Jung et al., group. Their behavioral battery began with the stressful Morris water maze testing and T-maze food restriction, while ours followed the standard order of less stressful to most [12, 78]. In addition, our sample size was twice the sample size per sex than the Jung study. Behavioral differences could also be attributed to background strain effects [79]. The hallmark characteristic that appears to define the *Arid1b*^+/−^ mice is a developmental delay, as evidenced by an overall total brain volume loss throughout development, and reduced body weight, length, and head width seen as early as PND8. Thus, stunted growth and failure to thrive seen in clinical populations is robustly recapitulated in our model. The cerebellum and hippocampus are also affected throughout development, showing both a genotype effect, a sex by genotype interaction, and an age by genotype interaction. Interestingly, in the adult mice, this hippocampal finding is absent, suggesting that while the developmental pattern of the hippocampus is affected, it does return to the appropriate size by adulthood. The effect of this altered development warrants further investigation since underlying differences in adulthood may be undetected at the mesoscopic scale of MRI examined here. However, in learning and memory behavioral tasks, *Arid1b* mutants did not show any deficits in hippocampal memory tasks as adults, suggesting functional effects, if any, do not persist.

## Supplementary Information

**Additional file 1:** Differentially expressed genes measured with an exploratory transcriptome analysis (RNA sequencing).

**Additional file 2:** Summary table for the behavioural statistics.

**Additional file 3:** Summary table for the supplemental behavioural statistics.

**Additional file 4:** Summary table for the negative behavioural statistics.

**Additional file 5:** Summary table of the Genotype, Genotype by Age, Genotype by Sex, and Genotype by Age by Sex Effects for the regional differences in the in vivo dataset.

**Additional file 6:** Summary table of the absolute and relative volume differences across the regions in the full group (males and females) and males and females separately.

## Data Availability

The datasets used and/or analysed during the current study are available from the corresponding author on reasonable request. Additionally, The neuroimaging data will be released at https://www.braincode.ca/ after the publication of this article.

## Abbreviations

Arid1b: AT-rich interactive domain 1B
ASD: Autism spectrum disorder
USV: Ultrasonic vocalizations
NDD: Neurodevelopmental disorder
ID: Intellectual disability
SFARI: Simons Foundation Autism Research Initiative
CSS: Coffin–Siris syndrome
MRI: Magnetic resonance imaging
MEMRI: Manganese-enhanced MRI
TCP: The Centre for Phenogenomics
MICe: Mouse Imaging Centre
CMMR: Canadian Mutant Mouse Repository
WT: Wild type
qRT-PCR: Quantitive reverse transcription polymerase chain reaction
GO: Gene ontology
T2: Transverse relaxation time
TR: Repetition time
TE: Echo time
PND: Postnatal day
ANTS: Advanced normalization tools
NOR: Novel object recognition
PTZ: Pentylenetetrazol
