# Supplementary material for: Neuroanatomy and behavior in mice with a haploinsufficiency of AT-rich interactive domain 1B (ARID1B) throughout development
Source: Mol Autism. 2021 Mar 23;12:25. doi: 10.1186/s13229-021-00432-y (PMC7986278; doi:10.1186/s13229-021-00432-y)
Supplement: Supplementary file 2 — Additional file 2: Summary table for the behavioural statistics. [file 13229_2021_432_MOESM2_ESM.docx]

| **Test** | **# of Animals** | **Metric** | **Time Point** | **Statistical Test** | **Statistic** | **p value** | **post hoc test** | **p value** |
| --- | --- | --- | --- | --- | --- | --- | --- | --- |
| Ultrasonic Vocalizations | ***Arid1b+/+*** N=40  ***Arid1b+/-*** N=19 | Ultrasonic Vocalizations Over Time | 3 | Two Way Repeated Measures ANOVA | Genotype F (1,57) = 6.665  Time F (3,171) = 6.679  Interaction F (3,171) = 2.68 | **p=.0124***  **p =.0003***  **p =.0486*** | ***Arid1b+/+*** vs  ***Arid1b+/-*** | **p =0.0020*** |
|  |  |  | 5 |  |  |  |  | p= 0.1575 |
|  |  |  | 7 |  |  |  |  | p = 0.9965 |
|  |  |  | 9 |  |  |  |  | p= 0.8945 |
|  |  | Sum of Ultrasonic Vocalizations |  | Unpaired Two-Tailed  T-Test | T (58) = 3.076 | **p=.003*** | -- | -- |
|  |  | Temperature Over Time | 3 | Two Way Repeated Measures ANOVA | Genotype F (1,57) = 3.919  Age F (3,171) = 20.68  Interaction F (3,171) = 0.112 | p=.0526  **p <.0001***  p=.9530 | ***Arid1b+/+*** vs  ***Arid1b+/-*** | p=.7303 |
|  |  |  | 5 |  |  |  |  | p=.5038 |
|  |  |  | 7 |  |  |  |  | p=.3077 |
|  |  |  | 9 |  |  |  |  | p=.6786 |
| Developmental Milestones | ***Arid1b+/+*** N=29  ***Arid1b+/-*** N=18 | Weight | 2 | Two Way Repeated Measures ANOVA | Genotype F (1,45) = 14.75  Age F (5,225) = 905.4  Interaction F (5,225) = 11.09 | **p=.0004***  **p <.0001***  **p<.0001*** | ***Arid1b+/+*** vs  ***Arid1b+/-*** | p=.9574 |
|  |  |  | 4 |  |  |  |  | p=.6335 |
|  |  |  | 6 |  |  |  |  | p=.4216 |
|  |  |  | 8 |  |  |  |  | **p=.0045*** |
|  |  |  | 10 |  |  |  |  | **p<.0001*** |
|  |  |  | 12 |  |  |  |  | **p<.0001*** |
|  |  | Body Length | 2 | Two Way Repeated Measures ANOVA | Genotype F (1,45) = 14.07  Age F (5,225) = 9081  Interaction F (5,225) = 4.571 | **p=.0005***  **p<.0001***  **p=.0005*** | ***Arid1b+/+*** vs  ***Arid1b+/-*** | p=.9038 |
|  |  |  | 4 |  |  |  |  | p=.2560 |
|  |  |  | 6 |  |  |  |  | p=.1172 |
|  |  |  | 8 |  |  |  |  | p=.3603 |
|  |  |  | 10 |  |  |  |  | **p=.005*** |
|  |  |  | 12 |  |  |  |  | **p<.0001*** |
|  |  | Head Width | 2 | Two Way Repeated Measures ANOVA | Genotype F (1,45) = 8.105  Age F (5,225) = 373.2  Interaction F (5,225) = 1.55 | **p=.0066***  **p<.0001***  p=.1753 | ***Arid1b+/+*** vs  ***Arid1b+/-*** | p=.3345 |
|  |  |  | 4 |  |  |  |  | p=.9172 |
|  |  |  | 6 |  |  |  |  | **p=.0460*** |
|  |  |  | 8 |  |  |  |  | p=.6803 |
|  |  |  | 10 |  |  |  |  | p=.9999 |
|  |  |  | 12 |  |  |  |  | **p=.0091*** |
|  |  | Righting Reflex | 2 | Two Way Repeated Measures ANOVA | Genotype F (1,45) = 3.71  Age F (5,225) = 48.85  Interaction F (5,225) = 3.448 | p=.0604  **p<.0001***  **p=.005*** | ***Arid1b+/+*** vs  ***Arid1b+/-*** | p=.0689 |
|  |  |  | 4 |  |  |  |  | **p=.004*** |
|  |  |  | 6 |  |  |  |  | p=.6820 |
|  |  |  | 8 |  |  |  |  | p=.9534 |
|  |  |  | 10 |  |  |  |  | p=.9999 |
|  |  |  | 12 |  |  |  |  | p=.9999 |
|  |  | Negative Geotaxis | 2 | Two Way Repeated Measures ANOVA | Genotype F (1,45) = 4.541  Age F (5,225) = 16.02  Interaction F (5, 225) = 1.993 | **p=.0385***  **p<.0001***  **p=.0806** | ***Arid1b+/+*** vs  ***Arid1b+/-*** | p=.9818 |
|  |  |  | 4 |  |  |  |  | **p=.0176*** |
|  |  |  | 6 |  |  |  |  | p=.9572 |
|  |  |  | 8 |  |  |  |  | p=.4789 |
|  |  |  | 10 |  |  |  |  | p=.9999 |
|  |  |  | 12 |  |  |  |  | p=.9999 |
|  |  | Circle Transverse | 6 | Two Way Repeated Measures ANOVA | Genotype F (1,45) = 4.344  Age F (3,135) = 28.89  Interaction F (3,135) = 0.403 | **p=.0429***  **p<.0001***  p=.7513 | ***Arid1b+/+*** vs  ***Arid1b+/-*** | p=.5994 |
|  |  |  | 8 |  |  |  |  | p=.2924 |
|  |  |  | 10 |  |  |  |  | p=.9988 |
|  |  |  | 12 |  |  |  |  | p=.8075 |
| Weight | ***Arid1b+/+*** N=29  ***Arid1b+/-*** N=18 | Weight | 65 | Two Way Repeated Measures ANOVA | Genotype F (1,51) = 15.22  Age F (2,102) = 92.53  Interaction F (2,102) = 6.268 | **p=.0003***  **p<.0001***  **p=.0027*** | ***Arid1b+/+*** vs  ***Arid1b+/-*** | **p=.0260*** |
|  |  |  | 100 |  |  |  |  | **p<.0001*** |
|  |  |  | 135 |  |  |  |  | **p=.0002*** |
| Grip Strength | ***Arid1b+/+*** N=28  ***Arid1b+/-*** N=25 | Maximum Forelimb Grip Strength |  | Unpaired Two-Tailed  T-Test | T (51) = 3.079 | **p=.0033*** |  |  |
| Automated Gait Analysis | ***Arid1b+/+*** N=21  ***Arid1b+/-*** N=19 | Stride Length | Forepaw | Two Way Repeated Measures ANOVA | Genotype F (1,42) = 3.094  Paw F (1,42) = 0.0201  Interaction F (1,42) = 0.3219 | p=.0859  p=.8879  p=.5735 | ***Arid1b+/+*** vs  ***Arid1b+/-*** | p=.8236 |
|  |  |  | Hindpaw |  |  |  |  | p=.6999 |
|  |  | Stride Frequency | Forepaw | Two Way Repeated Measures ANOVA | Genotype F (1,43) = 2.593  Paw F (1,43) = 0.1064  Interaction F (1,43) = 0.6412 | p=.1146  p=.7458  p=.4277 | ***Arid1b+/+*** vs  ***Arid1b+/-*** | p=.1712 |
|  |  |  | Hindpaw |  |  |  |  | p=.1532 |
| Open Field | ***Arid1b+/+*** N=28  ***Arid1b+/-*** N=25 | Horizontal Activity Over Time | 1-5 | Two Way Repeated Measures ANOVA | Genotype F (1,52) = 14.71  Time F (5, 260) = 42.02  Interaction F (5,260) = 0.8905 | **p =.0003***  **p<.0001***  p=.4879 | ***Arid1b+/+*** vs  ***Arid1b+/-*** | **p=.0463*** |
|  |  |  | 6-10 |  |  |  |  | **p=.0399*** |
|  |  |  | 11-15 |  |  |  |  | **p=.0280*** |
|  |  |  | 16-20 |  |  |  |  | **p=.0057*** |
|  |  |  | 21-25 |  |  |  |  | **p=.0005*** |
|  |  |  | 26-30 |  |  |  |  | **p=.0011*** |
|  |  | Vertical Activity Over Time | 1-5 | Two Way Repeated Measures ANOVA | Genotype F (1,52) = 2.802  Time F (5,260) = 26.06  Interaction F (5,260) = 1.433 | **p=.1001**  **p<.0001***  p=.2127 | ***Arid1b+/+*** vs  ***Arid1b+/-*** | p=.3074 |
|  |  |  | 6-10 |  |  |  |  | p=.9761 |
|  |  |  | 11-15 |  |  |  |  | p=.9087 |
|  |  |  | 16-20 |  |  |  |  | p=.7739 |
|  |  |  | 21-25 |  |  |  |  | p=.1525 |
|  |  |  | 26-30 |  |  |  |  | p=.3022 |
|  |  | Total Activity Over Time | 1-5 | Two Way Repeated Measures ANOVA | Genotype F (1,52) = 20.96  Time F (5, 260) = 52.2  Interaction F (5,260) = 0.2491 | **p<.0001***  **p<.0001***  p=.9400 | ***Arid1b+/+*** vs  ***Arid1b+/-*** | **p=.0015*** |
|  |  |  | 6-10 |  |  |  |  | **p=.0040*** |
|  |  |  | 11-15 |  |  |  |  | **p=.0082*** |
|  |  |  | 16-20 |  |  |  |  | **p=.0077*** |
|  |  |  | 21-25 |  |  |  |  | **p=.0016*** |
|  |  |  | 26-30 |  |  |  |  | **p=.0003*** |
|  |  | Sum Total Activity |  | Unpaired Two-Tailed  T-Test | T (52) =4.578 | **p<.0001*** |  |  |
| Social Approach | ***Arid1b+/+*** N=28  ***Arid1b+/-*** N=25 | Time in Chamber | Novel Object | Two Way Repeated Measures ANOVA | Genotype F (1,51) = 3.016  Chamber F (1,51) = 103.6  Interaction F (1,51) = 1.467 | p=.0885  **p<.0001***  p=.2313 | *Novel Object vs. Novel Mouse* | *Arid1b+/+* **p<.0001*** |
|  |  |  | Novel Mouse |  |  |  |  | *Arid1b+/-* **p<.0001*** |
|  |  | Time Sniffing | Novel Object | Two Way Repeated Measures ANOVA | Genotype F (1,51) = 0.03163  Chamber F (1,51) = 127.5  Interaction F (1,51) = 1.244 | p=.8595  **p<.0001***  p=.2699 | *Novel Object vs. Novel Mouse* | *Arid1b+/+* **p<.0001*** |
|  |  |  | Novel Mouse |  |  |  |  | *Arid1b+/-* **p<.0001*** |
|  |  | Transitions | Novel Object | Two Way Repeated Measures ANOVA | Genotype F (1,51) = 8.486  Chamber F (1,51) = 0.08332  Interaction F (1,51) = 0.1022 | **p=.005***  p=.7740  p=.7506 | ***Arid1b+/+*** vs  ***Arid1b+/-*** | p=.0765 |
|  |  |  | Novel Mouse |  |  |  |  | p=.2142 |
| Male Female Reciprocal Interaction | ***Arid1b+/+*** N=15  ***Arid1b+/-*** N=17 | Nose-to-Anogenital Sniffing |  | Unpaired Two-Tailed  T-Test | T (30) = 1.967 | **p=.05*** |  |  |
|  |  | Following |  | Unpaired Two-Tailed  T-Test | T (30) = 2.266 | **p=.0308*** |  |  |
|  |  | Sum USVs |  | Unpaired Two-Tailed  T-Test | T (29) =2.119 | **p=.0428*** |  |  |
| Self-Groom | ***Arid1b+/+*** N=27  ***Arid1b+/-*** N=25 | Time Grooming |  | Unpaired Two-Tailed  T-Test | T (50) = 0.8209 | p=.4156 |  |  |
| Elevated Plus Maze | ***Arid1b+/+*** N=28  ***Arid1b+/-*** N=27 | %Time in Open Arm |  | Unpaired Two-Tailed  T-Test | T (53) = 2.101 | **p=.0404*** |  |  |
|  |  | Total Entries |  | Unpaired Two-Tailed  T-Test | T (53) = 4.609 | **p<.0001*** |  |  |
| Seizure Susceptibility | ***Arid1b+/+*** N=28  ***Arid1b+/-*** N=25 | Latency to Loss of Righting |  | Unpaired Two-Tailed  T-Test | T (83) = 2.491 | **p=.0147*** |  |  |
|  |  | Latency to Tonic Clonic Seizure |  | Unpaired Two-Tailed  T-Test | T (67) = 2.114 | **p=.0383*** |  |  |
|  |  | Latency to Death |  | Unpaired Two-Tailed  T-Test | T (77) = 2.553 | **p=.0126*** |  |  |
| Touchscreen-  Pairwise Discrimination | ***Arid1b+/+*** N=24  ***Arid1b+/-*** N=15 | Sessions to Criteria |  | Unpaired Two-Tailed  T-Test | T (37) =3.073 | **p=.004*** |  |  |
|  |  | % Animals Completed |  | Gehan Breslow Wilcoxon Chi Square Test | X = 8.876 | **p=.0029*** |  |  |
| Touchscreen-  Reversal of  Pairwise Discrimination | ***Arid1b+/+*** N=20  ***Arid1b+/-*** N=14 | Sessions to Criteria |  | Unpaired Two-Tailed  T-Test | T (32) = 1.921 | p=.0637 |  |  |
|  |  | % Animals Completed |  | Gehan Breslow Wilcoxon Chi Square Test | X = 2.572 | p=.1088 |  |  |
| Fear Conditioning | ***Arid1b+/+*** N=27  ***Arid1b+/-*** N=24 | Time spent freezing | Pre-Training | Two Way Repeated Measures ANOVA | Genotype F (1,48) = 1.738  Training F (1,48) = 108.9  Interaction F (1,48) = 3.024 | p=.1935  **p<.0001***  p=.0885 | Pre-Training vs Post Training | *Arid1b+/+* p=.9699 |
|  |  |  | Post Training |  |  |  |  | *Arid1b+/-* p=.0670 |
|  |  |  | Contextual | Unpaired Two-Tailed  T-Test | T (49) = 0.2059 | p=.8377 |  |  |
|  |  |  | Pre-Cue | Two Way Repeated Measures ANOVA | Genotype F (1,48) = 2.864  Cue F (1,48) = 205.4  Interaction F (1,48) = 0.8087 | p=.0971  **p<.0001***  p=.3730 | Pre-Cue vs Post Cue | *Arid1b+/+* **p<.0001*** |
|  |  |  | Post Cue |  |  |  |  | *Arid1b+/-* **p<.0001*** |
| Novel Object Recognition | ***Arid1b+/+*** N=27  ***Arid1b+/-*** N=21 | Time sniffing | Familiar Object | Two Way Repeated Measures ANOVA | Genotype F (1,46) = 1.717  Object F (1,46) = 12.36  Interaction F (1,46) = 0.3488 | p=.1966  **p=.001***  p=.5577 | Familiar Object vs Novel Object | *Arid1b+/+* **p=.0065*** |
|  |  |  | Novel Object |  |  |  |  | *Arid1b+/-* p=.1113 |
