# Supplementary material for: Neuroanatomy and behavior in mice with a haploinsufficiency of AT-rich interactive domain 1B (ARID1B) throughout development
Source: Mol Autism. 2021 Mar 23;12:25. doi: 10.1186/s13229-021-00432-y (PMC7986278; doi:10.1186/s13229-021-00432-y)
Supplement: Supplementary file 3 — Additional file 3: Summary table for the supplemental behavioural statistics. [file 13229_2021_432_MOESM3_ESM.docx]

| **Test** | **# of Animals** | **Metric** | **Time Point** | **Statistical Test** | **Statistic** | **p value** | **post hoc test** | **p value** |
| --- | --- | --- | --- | --- | --- | --- | --- | --- |
| Developmental Milestones | ***Arid1b+/+*** N=29  ***Arid1b+/-*** N=18 | Cliff Aversion | 2 | Two Way Repeated Measures ANOVA | Genotype F (1,45) = 0.438  Age F (5,225) = 16.48  Interaction F (5,225) = 0.2884 | p=.5115  **p <.0001***  p=.9191 | ***Arid1b+/+*** vs  ***Arid1b+/-*** | p>.9999 |
|  |  |  | 4 |  |  |  |  | p=.9998 |
|  |  |  | 6 |  |  |  |  | p=.7785 |
|  |  |  | 8 |  |  |  |  | p=.9947 |
|  |  |  | 10 |  |  |  |  | p>.9999 |
|  |  |  | 12 |  |  |  |  | p>.9999 |
|  |  | Forelimb Hang | 2 | Two Way Repeated Measures ANOVA | Genotype F (1,45) = 0.6008  Age F (5,225) = 38.03  Interaction F (5,225) = 0.4162 | p=.4423  **p<.0001***  p=.8372 | ***Arid1b+/+*** vs  ***Arid1b+/-*** | p>.9999 |
|  |  |  | 4 |  |  |  |  | p>.9999 |
|  |  |  | 6 |  |  |  |  | p=.9919 |
|  |  |  | 8 |  |  |  |  | p=.9452 |
|  |  |  | 10 |  |  |  |  | p=.9962 |
|  |  |  | 12 |  |  |  |  | p=.8282 |
|  |  | Hindlimb Hang | 2 | Two Way Repeated Measures ANOVA | Genotype F (1,45) = 3.171  Age F (5,225) = 6.851  Interaction F (5,225) = 3.099 | p=.0817  **p<.0001***  p=.0100* | ***Arid1b+/+*** vs  ***Arid1b+/-*** | p=.2180 |
|  |  |  | 4 |  |  |  |  | p=.6263 |
|  |  |  | 6 |  |  |  |  | p>.9999 |
|  |  |  | 8 |  |  |  |  | p=.2009 |
|  |  |  | 10 |  |  |  |  | p=.2870 |
|  |  |  | 12 |  |  |  |  | p=.2191 |
| Open Field | ***Arid1b+/+*** N=28  ***Arid1b+/-*** N=25 | Center Time Over Time | 1-5 | Two Way Repeated Measures ANOVA | Genotype F (1,52) = 0.1754  Time F (5, 260) = 2.808  Interaction F (5,260) = 2.461 | **p =.6770**  **p=.0173***  **p=.0335*** | ***Arid1b+/+*** vs  ***Arid1b+/-*** | p=.0570 |
|  |  |  | 6-10 |  |  |  |  | p=.9852 |
|  |  |  | 11-15 |  |  |  |  | p>.9999 |
|  |  |  | 16-20 |  |  |  |  | p=.9759 |
|  |  |  | 21-25 |  |  |  |  | p=.9980 |
|  |  |  | 26-30 |  |  |  |  | p>.9999 |
| Social Approach  Familiarization | ***Arid1b+/+*** N=28  ***Arid1b+/-*** N=25 | Time in Chamber | Top Chamber | Two Way Repeated Measures ANOVA | Genotype F (1,51) = .0001  Chamber F (2, 102) = 28.98  Interaction F (2,102) = 1.468 | p=.9916  **p<.0001***  p=.2123 | ***Arid1b+/+*** vs  ***Arid1b+/-*** | p=.3710 |
|  |  |  | Middle Chamber |  |  |  |  | p>.9999 |
|  |  |  | Bottom Chamber |  |  |  |  | p=.3803 |
|  |  | Transitions | Top Chamber | Two Way Repeated Measures ANOVA | Genotype F (1,51) = 18.93  Chamber F (1,51) = 12.9  Interaction F (1.48) = 0.05771 | **p<.0001***  **p=.0007***  p=.8111 | ***Arid1b+/+*** vs  ***Arid1b+/-*** | **p=.0002*** |
|  |  |  | Bottom Chamber |  |  |  |  | **p=.0004*** |
| Male Female Reciprocal Interaction | ***Arid1b+/+*** N=15  ***Arid1b+/-*** N=17 | Time Nose-to-Nose Sniffing |  | Unpaired Two-Tailed  T-Test | T (30) = 0.6011 | p=.5523 |  |  |
|  |  | Time Exploring |  | Unpaired Two-Tailed  T-Test | T (30) = 1.941 | p=.0617 |  |  |
|  |  | Time Grooming |  | Unpaired Two-Tailed  T-Test | T (29) = 0.5457 | p=.5893 |  |  |
| Novel Object Recognition  Familiarization | ***Arid1b+/+*** N=28  ***Arid1b+/-*** N=25 | Time sniffing |  | Two Way Repeated Measures ANOVA | Genotype F (1,51) = 1.52  Object F (1,51) = 6.493  Interaction F (1,51) = 0.2141 | p=.2233  **p=.0140***  p=.6456 | Right vs Left Object | *Arid1b+/+* p=.2451 |
|  |  |  |  |  |  |  |  | *Arid1b+/-* p=.0889* |
