# Supplementary material for: Neuroanatomy and behavior in mice with a haploinsufficiency of AT-rich interactive domain 1B (ARID1B) throughout development
Source: Mol Autism. 2021 Mar 23;12:25. doi: 10.1186/s13229-021-00432-y (PMC7986278; doi:10.1186/s13229-021-00432-y)
Supplement: Supplementary file 4 — Additional file 4: Summary table for the negative behavioural statistics. [file 13229_2021_432_MOESM4_ESM.docx]

| **Test** | **# of Animals** | **Metric** | **Time Point** | **Statistical Test** | **Statistic** | **p value** | **post hoc test** | **p value** |
| --- | --- | --- | --- | --- | --- | --- | --- | --- |
| Developmental Milestones | ***Arid1b+/+*** N=29  ***Arid1b+/-*** N=18 | Cliff Aversion | 2 | Two Way Repeated Measures ANOVA | Genotype F (1,45) = 0.438  Age F (5,225) = 16.48  Interaction F (5,225) = 0.2884 | p=.5115  **p <.0001***  p=.9191 | ***Arid1b+/+*** vs  ***Arid1b+/-*** | p>.9999 |
|  |  |  | 4 |  |  |  |  | p=.9998 |
|  |  |  | 6 |  |  |  |  | p=.7785 |
|  |  |  | 8 |  |  |  |  | p=.9947 |
|  |  |  | 10 |  |  |  |  | p>.9999 |
|  |  |  | 12 |  |  |  |  | p>.9999 |
|  |  | Forelimb Hang | 2 | Two Way Repeated Measures ANOVA | Genotype F (1,45) = 0.6008  Age F (5,225) = 38.03  Interaction F (5,225) = 0.4162 | p=.4423  **p<.0001***  p=.8372 | ***Arid1b+/+*** vs  ***Arid1b+/-*** | p>.9999 |
|  |  |  | 4 |  |  |  |  | p>.9999 |
|  |  |  | 6 |  |  |  |  | p=.9919 |
|  |  |  | 8 |  |  |  |  | p=.9452 |
|  |  |  | 10 |  |  |  |  | p=.9962 |
|  |  |  | 12 |  |  |  |  | p=.8282 |
|  |  | Hindlimb Hang | 2 | Two Way Repeated Measures ANOVA | Genotype F (1,45) = 3.171  Age F (5,225) = 6.851  Interaction F (5,225) = 3.099 | p=.0817  **p<.0001***  p=.0100* | ***Arid1b+/+*** vs  ***Arid1b+/-*** | p=.2180 |
|  |  |  | 4 |  |  |  |  | p=.6263 |
|  |  |  | 6 |  |  |  |  | p>.9999 |
|  |  |  | 8 |  |  |  |  | p=.2009 |
|  |  |  | 10 |  |  |  |  | p=.2870 |
|  |  |  | 12 |  |  |  |  | p=.2191 |
| Spontaneous Alternation | ***Arid1b+/+*** N=28  ***Arid1b+/-*** N=25 | % Spontaneous Alternations |  | Unpaired Two-Tailed  T-Test | T (51) = 1.63 | p=.1092 |  |  |
| Open Field | ***Arid1b+/+*** N=28  ***Arid1b+/-*** N=25 | Center Time Over Time | 1-5 | Two Way Repeated Measures ANOVA | Genotype F (1,52) = 0.1754  Time F (5, 260) = 2.808  Interaction F (5,260) = 2.461 | **p =.6770**  **p=.0173***  **p=.0335*** | ***Arid1b+/+*** vs  ***Arid1b+/-*** | p=.0570 |
|  |  |  | 6-10 |  |  |  |  | p=.9852 |
|  |  |  | 11-15 |  |  |  |  | p>.9999 |
|  |  |  | 16-20 |  |  |  |  | p=.9759 |
|  |  |  | 21-25 |  |  |  |  | p=.9980 |
|  |  |  | 26-30 |  |  |  |  | p>.9999 |
| Light Dark Conflict | ***Arid1b+/+*** N=15  ***Arid1b+/-*** N=17 | Time in Chamber |  | Unpaired Two-Tailed  T-Test | T (52) = 1.438 | p=.1565 |  |  |
|  |  | Transitions |  | Unpaired Two-Tailed  T-Test | T (52) = 0.7623 | p=.4493 |  |  |
|  |  | Latency to Dark Transition |  | Unpaired Two-Tailed  T-Test | T (52) = 0.4908 | p=.6257 |  |  |
| Hot Plate | ***Arid1b+/+*** N=27  ***Arid1b+/-*** N=25 | Latency to Response |  | Unpaired Two-Tailed  T-Test | T (50) = 0.5626 | p=.5762 |  |  |
